# Metabolic strategies that enable oral commensal persistence in a lower airway environment

**DOI:** 10.1128/mbio.01948-25

**Published:** 2025-09-22

**Authors:** Ashley M. Toney, Junu Koirala, Apollo Stacy

**Affiliations:** 1 Department of Cardiovascular and Metabolic Sciences, Cleveland Clinic Research, Cleveland, Ohio, USA; 2 Department of Molecular Medicine, Cleveland Clinic Lerner College of Medicine, Case Western Reserve University School of Medicine 12304, Cleveland, Ohio, USA; University of Louisville, Louisville, Kentucky, USA

**Keywords:** bronchiectasis, cystic fibrosis, sputum, *Neisseria*, transposon sequencing, nitrate respiration, antibiotic resistance, oral-lung axis

## Abstract

Oral microbiota are increasingly implicated in chronic inflammatory diseases beyond the mouth, including bronchiectasis, a condition marked by persistent airway inflammation, mucus accumulation, and limited therapeutic options. Among these microbes, commensal *Neisseria*, typically considered health associated in the upper airway, are emerging as opportunistic colonists of the inflamed lower airway. However, mechanisms supporting their persistence in this hostile environment, characterized by antibiotic pressure, nutrient limitation, and interspecies competition, remain poorly defined. Here, we show that the prevalent oral commensal *Neisseria mucosa* exhibits markedly enhanced antibiotic resistance and anoxic growth in a synthetic medium (SCFM2) that mimics sputum from individuals with bronchiectasis. Using genome-wide transposon sequencing, we identified key genetic determinants of *N. mucosa* fitness, including pathways for L-lactate catabolism and pyrimidine biosynthesis. Notably, nitrate respiration was also essential for growth, linking use of this inflammation-associated electron acceptor to fitness under oxygen-limited, sputum-like conditions. Loss of nitrate reductase impaired anoxic L-lactate catabolism, reduced competitive fitness against *Pseudomonas aeruginosa*, and abrogated growth in SCFM2, as well as human saliva—a relevant, nitrate-rich nutrient source in the oral cavity. Furthermore, chemical inhibition of nitrate reductase using tungstate suppressed growth of both *N. mucosa* and *P. aeruginosa* in SCFM2, but not in standard media, revealing a context-specific metabolic vulnerability of nitrate-respiring airway pathogens. These findings suggest that inflammation-compatible traits like nitrate respiration, while supporting oral commensalism, may also drive opportunistic expansion in the inflamed airway. Targeting such pathways could offer a non-antibiotic approach to limit oral bacterial persistence in bronchiectasis and other chronic diseases.

**IMPORTANCE:**

Chronic respiratory diseases such as bronchiectasis and cystic fibrosis are marked by persistent infection and inflammation, with oral bacteria increasingly recognized as active contributors. Among these, *Neisseria* species, typically considered health-associated commensals, are frequently detected in the lower airway, yet little is known about how they persist in this hostile environment. Here, we identify key metabolic pathways that support the survival of *Neisseria mucosa* in sputum-like media, including nitrate respiration, a pathway closely linked to inflammation. We also show that *N. mucosa* exhibits enhanced antibiotic resistance under these conditions, underscoring the need to study microbial physiology in host-relevant contexts. Finally, we demonstrate that a selective inhibitor of nitrate respiration suppresses *N. mucosa* growth in sputum-like but not standard media, revealing a context-specific vulnerability. These findings suggest that targeting inflammation-compatible metabolic pathways may inform new, non-antibiotic approaches for managing chronic respiratory diseases.

## INTRODUCTION

The human oral cavity harbors one of the most taxonomically rich microbial communities in the body, comprising over 800 described taxa (1). Although oral microbiota are generally commensal and spatially confined to the upper aerodigestive tract, a growing body of research suggests that they can contribute to disease far beyond their primary habitat (2). In addition to local conditions, such as dental caries and periodontitis, oral microbes have been implicated in a broad range of distal diseases, including cardiovascular disease, diabetes, rheumatoid arthritis, and inflammatory bowel disease. These associations are thought to involve several indirect, long-range mechanisms, including endotoxemia (3), circulation of microbially produced metabolites (4, 5), and maladaptive trained immunity (6).

Another major route of oral microbiota-driven disease is the direct colonization of extraoral sites, particularly in individuals with compromised immunity. While typically benign, oral commensals can translocate to distal niches, where they can exacerbate inflammation (7, 8) or establish opportunistic infections, such as brain abscesses (9), infective endocarditis (10, 11), and respiratory infections (12, 13). This is surprising, given that many oral taxa are nutritional specialists with reduced genomes and narrow ecological niches (14, 15), raising a fundamental question: how do oral-adapted microbes survive and even thrive in radically different environments across the human body?

One such environment is the chronically inflamed lower airway. While aspiration of oral contents has long been recognized as a cause of acute pneumonia (16), recent work has also implicated oral microbes in chronic respiratory diseases, including chronic obstructive pulmonary disease (COPD), bronchiectasis, and cystic fibrosis (CF) (17–20). While etiologically distinct, these conditions share in common persistent inflammation, impaired mucociliary clearance, and recurrent infection, which collectively remodel the airway microbiota (21). In CF and non-CF bronchiectasis, both of which are marked by irreversible airway dilation and mucus accumulation, oral taxa such as *Streptococcus*, *Prevotella*, and *Veillonella* are frequently detected in sputum and bronchoalveolar lavage samples (12, 22, 23). Though partly attributable to upper airway contamination (24), mounting evidence points toward a functional role for these taxa in shaping community structure, immune responses, and disease severity, similar to the contributions of canonical pathogens like *Pseudomonas aeruginosa* (12, 25–28).

Among oral taxa linked to bronchiectasis, commensal *Neisseria* are of particular interest. This complex genus of gram-negative, aerobic diplococci is notorious for the non-oral pathogens *N. gonorrhoeae* and *N. meningitidis*, causative agents of gonorrhea and meningitis, respectively (29). At the same time, the genus comprises several distinct commensal species, including *N. cinerea*, *N. elongata*, *N. lactamica*, *N. mucosa*, *N. sicca*, and *N. subflava* (30, 31). Traditionally regarded as health-associated members of the upper airway microbiota, these taxa contribute to microbial community stability (32, 33) and can modulate host immune responses (34, 35). Within the oral cavity, they can exhibit biogeographic niche specialization, with some species showing preference for specific habitats such as the tongue dorsum or dental plaque (14, 36). Notably, these spatial distributions are reflected in habitat-specific functional adaptations, with plaque-associated *Neisseria* (e.g., *N. mucosa*) showing enhanced capability for nitrogen metabolism (14).

However, recent studies have reported *Neisseria* enrichment in patients with chronic lung diseases, particularly bronchiectasis (12, 13, 21–23, 37), raising questions about their potential role in disease progression. While not considered primary pathogens, the fact that commensal *Neisseria* (i) can persist in inflamed airways, (ii) exacerbate pulmonary disease in animal models, and (iii) share key metabolic and virulence-related traits with pathogenic *Neisseria* (e.g., type IV pili) suggests that they function as opportunistic pathobionts or commensals capable of contributing to disease under permissive environmental conditions (12, 38–41). But despite their emerging clinical relevance, the physiological requirements that enable *Neisseria* to colonize the lower airway remain poorly defined.

A central knowledge gap is whether oral *Neisseria* employ distinct metabolic strategies to persist within the altered nutritional landscape of the inflamed lung. In CF and non-CF bronchiectasis, the lower airways are replete with host-derived substrates such as mucins, extracellular DNA, lactate, and nitrate, many of which accumulate as a consequence of chronic inflammation and immune activation (42–47). While multiomics analyses have revealed community-wide metabolic shifts involving these metabolites, few studies have examined how individual taxa, particularly commensals, exploit these resources to support their fitness *in situ* (12, 48, 49). Moreover, given that inflammation can remodel the airway environment in predictable ways, understanding how oral commensals respond to these changes could uncover broadly relevant principles of microbial persistence and inform new therapeutic approaches beyond conventional antibiotics, which are increasingly limited due to rising resistance and off-target disruption of beneficial microbiota (50).

Here, we used *N. mucosa*, one of the most abundant and widespread species of oral *Neisseria* (14, 36), as a model organism to dissect the metabolic requirements for oral commensal survival in the lower airway. To this end, we leveraged synthetic cystic fibrosis medium (SCFM2), a highly validated, chemically defined medium (CDM) that mimics the nutrient composition of CF airway secretions (51–55). Using this *in vitro* system, we performed transposon sequencing (Tn-seq) (56) to identify genes required for *N. mucosa* fitness under anoxic, sputum-like conditions. This genome-wide screen revealed several critical metabolic functions, including L-lactate catabolism, pyrimidine biosynthesis, and nitrate respiration. Notably, while nitrate respiration is likely adaptive in inflamed contexts (57), including bronchiectasis (46, 58, 59), we show that it also supports *N. mucosa* growth in saliva, suggesting that commensal metabolic traits may be co-opted during opportunistic infection. Finally, we demonstrate that *N. mucosa* exhibits enhanced resistance to multiple, clinically relevant antibiotics when cultured in SCFM2, but remains susceptible to a selective inhibitor of nitrate respiration (60), highlighting the value of using disease-relevant media in therapeutic screening.

Together, our findings illuminate the metabolic logic by which a normally benign oral commensal can persist in the inflamed airway and underscore the therapeutic potential of targeting environment-specific microbial vulnerabilities in chronic lung disease.

## RESULTS

### Synthetic sputum enhances *N. mucosa* antibiotic resistance and anoxic growth

*Neisseria* species are common colonists of the upper respiratory tract, including the oral cavity, in healthy individuals. However, to persist in the lower respiratory tract, particularly in patients with chronic airway diseases, commensal *Neisseria* must withstand numerous host and environmental stressors, including antibiotic exposure. The macrolide azithromycin is one of the most widely prescribed antibiotics for bronchiectasis (61, 62). It was also once a frontline treatment for pathogenic *Neisseria* (e.g., *N. gonorrhoeae*), but is now largely avoided due to rising resistance, which can emerge in both pathogenic and commensal species (63, 64). While resistance is often linked to genetic mechanisms (e.g., target site mutation), non-heritable “phenotypic” antibiotic resistance, driven by environmental factors such as pH or nutrient availability, can also contribute to reduced susceptibility (65, 66).

**Fig 1:**
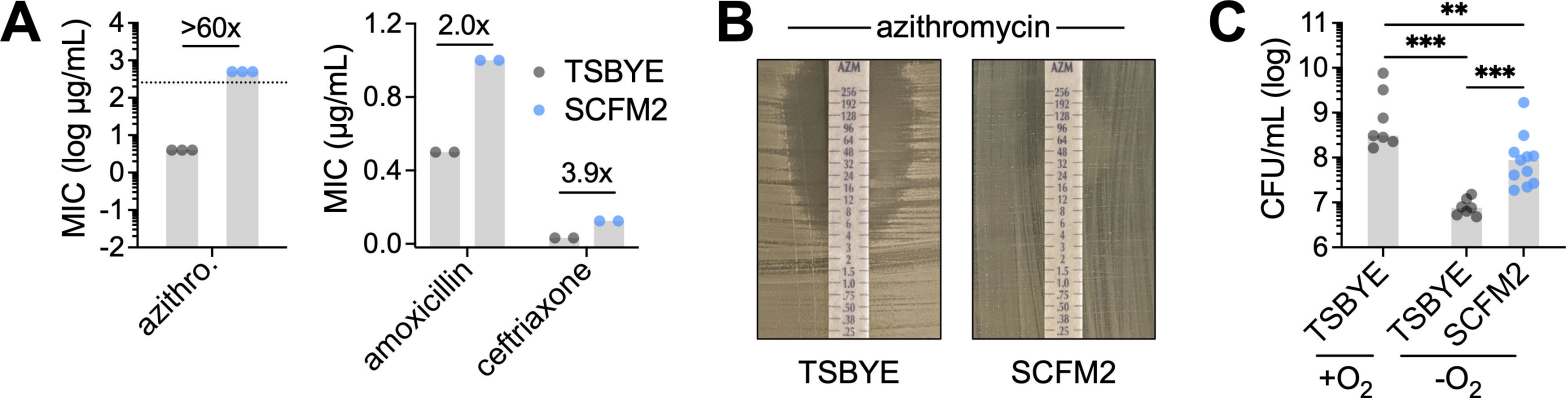
Synthetic sputum enhances *N. mucosa* antibiotic resistance and anoxic growth. (**A**) Minimum inhibitory concentrations of the indicated antibiotics for *N. mucosa* cultured as a lawn on tryptic soy broth + yeast extract or SCFM2 agar. Lawns were cultured under oxic conditions since growth was not perceptible under anoxic conditions. Data represent ≥2 biological replicates (performed on separate days); gray bars, median; numbers above bars, fold change in median. Dotted line (left panel) indicates the max MIC value. (**B**) Representative MIC results for azithromycin. (**C**) Growth yields of *N. mucosa* after culture for 24 h in TSBYE or SCFM2 liquid media under oxic (+O_2_) or anoxic (−O_2_) conditions. Media were not de-oxygenated prior to inoculation at OD_600_ = 0.001 (~10^5^ CFU/mL). Data represent ≥4 biological replicates, each with 1–2 technical replicates (total *n* = 7–11); gray bars, median; **, *P* < 0.01; ***, *P* < 0.001 (two-tailed Mann-Whitney test).

To explore this in *N. mucosa*, a common commensal and opportunistic lung colonist, we used SCFM2, which mimics the nutrient composition of sputum from individuals with cystic fibrosis, a condition often associated with bronchiectasis (51). Using a strain originally isolated from patient sputum (ATCC 19696 [67]), we determined the minimum inhibitory concentration (MIC) of azithromycin on SCFM2 and compared it to that on a standard nutrient-rich laboratory medium, tryptic soy broth supplemented with yeast extract (TSBYE). On TSBYE, the MIC of azithromycin was 4 µg/mL, consistent with reported values for resistant clinical isolates (68) (Fig. 1A and B). In contrast, the MIC on SCFM2 exceeded 256 µg/mL, indicating dramatically reduced susceptibility under sputum-like conditions (Fig. 1A and B).

To extend this analysis, we determined the MICs of two additional, clinically relevant antibiotics: the β-lactam amoxicillin, commonly used to treat bronchiectasis (69), and the cephalosporin ceftriaxone, a standard treatment for invasive *Neisseria* infections (63). In both cases, *N. mucosa* exhibited moderately increased resistance on SCFM2 compared to TSBYE (Fig. 1A), suggesting a broader pattern of phenotypic resistance in sputum-like environments.

Given that sputum is often anoxic (70, 71), we next examined the impact of oxygen limitation on *N. mucosa* growth. As expected based on the frequent classification of *Neisseria* as “aerobes” (72), *N. mucosa* exhibited a >40-fold lower growth yield under anoxic vs oxic conditions in TSBYE (Fig. 1C). In contrast, when cultured under the same anoxic conditions in SCFM2, *N. mucosa* achieved a final yield 12-fold higher than in TSBYE (Fig. 1C), suggesting improved adaptation to oxygen limitation.

Together, these findings indicate that sputum-like conditions modeled by SCFM2 promote both phenotypic antibiotic resistance and anoxic growth in *N. mucosa*. These results highlight how local environmental factors at the infection site can potentially enhance the fitness and persistence of commensal *Neisseria* in the lower respiratory tract.

### Tn-seq identifies *N. mucosa* fitness determinants in synthetic sputum

Given that SCFM2 promotes phenotypic antibiotic resistance in *N. mucosa*, we next sought to identify bacterial functions that are essential for growth in this sputum-like environment. In doing so, we aimed to uncover potential targets for novel, non-antibiotic therapies that could directly disrupt *N. mucosa* fitness in the lung.

To this end, we performed a genome-wide transposon (Tn) mutant screen, Tn-seq, to identify genes required for *N. mucosa* replication in SCFM2. A key advantage of Tn-seq is that it enables rapid quantification of relative Tn mutant abundance (fitness) across a pooled Tn mutant library by sequencing genomic regions adjacent to Tn insertions (56).

To generate a Tn mutant pool in *N. mucosa*, we leveraged its natural competence for transformation (73). Specifically, we mutagenized purified *N. mucosa* genomic DNA *in vitro* using EZ-Tn5 transposition (74) and then naturally transformed this DNA back into *N. mucosa* cells (see Materials and Methods). The resulting transformants were pooled and preserved as single-use aliquots. Analysis of three independent aliquots identified 33,568 unique high-confidence Tn insertions (i.e., present in all three replicates), corresponding to an average of one insertion every 80 bp across the ~2.7 Mbp *N. mucosa* 19696 genome.

**Fig 2:**
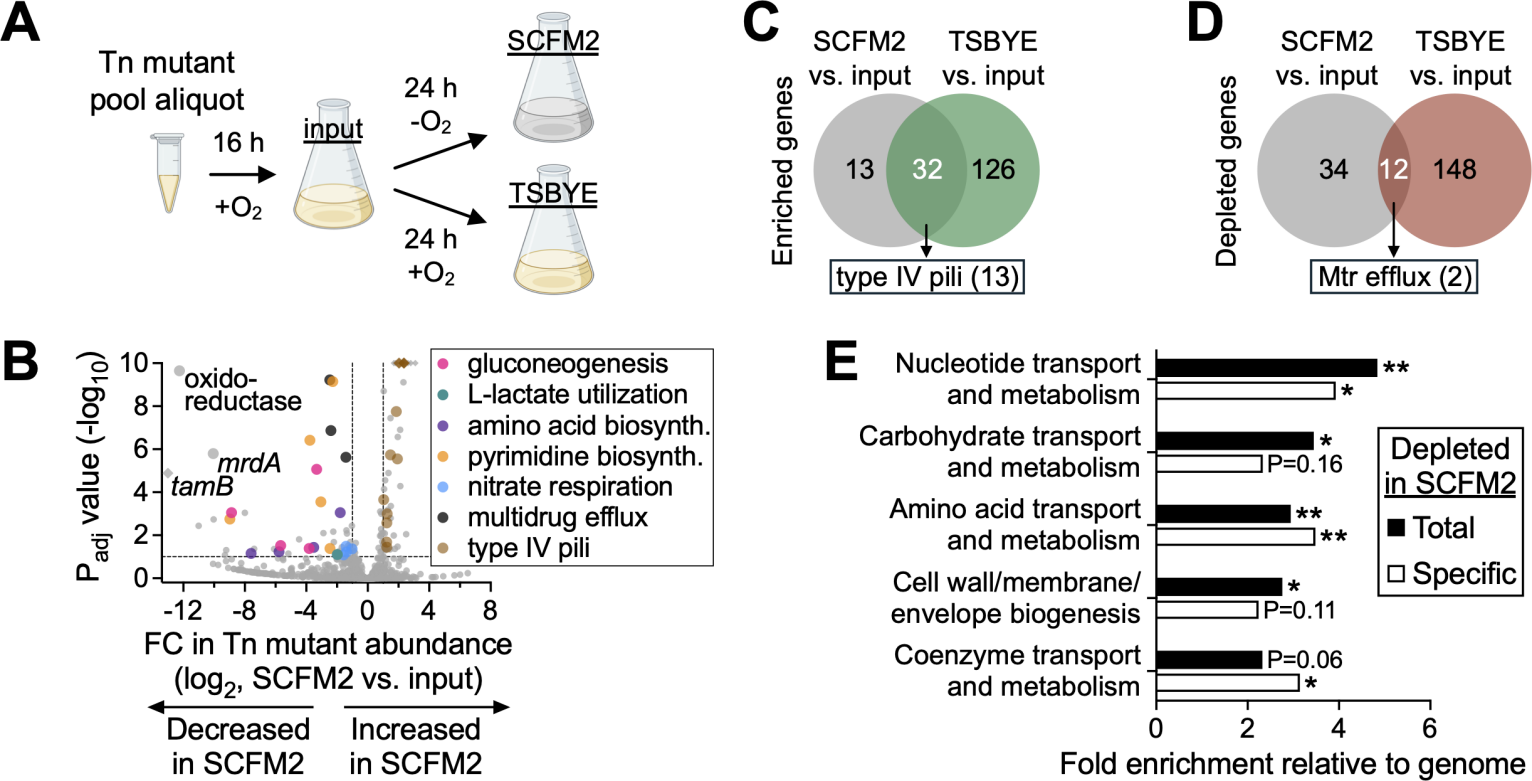
Tn-seq identifies *N. mucosa* fitness determinants in synthetic sputum. (**A**) Tn-seq experimental design. A cryo-aliquot of the *N. mucosa* Tn mutant pool was revived for 16–20 h in tryptic soy broth + yeast extract under oxic conditions (input condition), then back-diluted and cultured for 24 h in synthetic cystic fibrosis medium under anoxic conditions or tryptic soy broth + yeast extract + 10 mM NaCl under oxic conditions. Media for the TSBYE condition (same as Fig. 5B) were de-oxygenated prior to inoculation. (**B**) Differential abundance of *N. mucosa* Tn mutants after anoxic growth in SCFM2. Genes (points) are colored by functional category. The x-axis represents fold change (FC) in total Tn mutant abundance per gene (SCFM2 vs input); y-axis, adjusted *P* values (DESeq2; Wald test with Benjamini-Hochberg correction); dashed lines, significance cutoffs (FC >2; *P*_adj_ <0.1 [default DESeq2 setting]). For visual clarity, 13 off-axis values (diamonds) were adjusted to the plot’s maximum FC or *P* value. (**C and D**) Venn diagrams showing the overlap between the number of *N. mucosa* genes in which Tn mutant abundance was (**C**) enriched or (**D**) depleted in both SCFM2 and TSBYE when compared to the input. (**C**) Of the 32 overlapping enriched genes, 13 coded for components of type IV pili, while (**D**) of the 12 overlapping depleted genes, 2 coded for subunits of the Mtr efflux pump. (**E**) Enrichment of clusters of orthologous group (COG) categories among genes in which Tn mutant abundance was depleted following anoxic growth in SCFM2. The x-axis represents the fraction of the 46 total SCFM2-depleted genes (black), or the 34 SCFM2-specific depleted genes (white), in a COG category divided by the genome-wide fraction of genes in the same category. *, *P* < 0.05; **, *P* < 0.01 (hypergeometric test).

We next subjected the Tn mutant pool to anoxic growth in SCFM2 and performed Tn-seq (Fig. 2A). We chose anoxic growth based on studies showing that oxygen levels in patient sputum are markedly low (70, 71), likely supporting the persistence of strictly anaerobic oral taxa such as *Prevotella* and *Veillonella* (12, 22, 23). Thus, to better reflect the oxygen-limited environment encountered by oral commensals in the lower airway, we conducted Tn-seq in anoxic SCFM2 (Fig. 2A, SCFM2 condition). As controls, we profiled the Tn mutant pool (i) immediately prior to growth in SCFM2, following oxic revival in TSBYE (Fig. 2A, input condition) and (ii) after oxic passage in TSBYE (Fig. 2A, TSBYE condition), paralleling the anoxic SCFM2 condition. Oxic growth in TSBYE was necessary because *N. mucosa* grows poorly in this medium under anoxic conditions (Fig. 1C).

Comparison of Tn mutant abundances before and after growth in SCFM2 (input vs SCFM2) revealed 46 genes with significantly reduced representation (*P*_adj_ <0.1), suggesting that these genes are required for *N. mucosa* fitness in SCFM2 (Fig. 2B, left side of plot). Among these, three genes exhibited particularly strong depletion: (i) an oxidoreductase of unknown function, (ii) *tamB*, encoding a subunit of the translocation and assembly module (TAM) complex involved in outer membrane protein insertion (75), and (iii) *mrdA*, encoding a peptidoglycan transpeptidase essential for cell wall synthesis (76).

This comparison also identified 45 genes with significantly increased representation in SCFM2 (Fig. 2B, right side of plot). Notably, 13 of these genes corresponded to components of type IV pili, suggesting that pili may impair *N. mucosa* fitness under sputum-like conditions. However, a similar enrichment of pili mutants was observed following oxic growth in TSBYE (input vs TSBYE). In fact, 32 of the 45 genes enriched in SCFM2 were also enriched in TSBYE, including all 13 pili genes (Fig. 2C), suggesting that their apparent fitness benefit is likely an artifact of *in vitro* culture rather than a specific adaptation to SCFM2.

In contrast, most genes depleted in SCFM2 appeared to be condition specific, as only 12 of the 46 depleted genes were also depleted in TSBYE (Fig. 2D). Mutants in these 12 genes may therefore exhibit general growth defects, or alternatively, these genes may contribute to fitness in both environments. One example is the Mtr efflux pump: two of its three subunits were depleted in both SCFM2 and TSBYE, while the third was specific to SCFM2. This efflux system is well characterized in pathogenic *Neisseria* for its role in azithromycin resistance (77, 78), suggesting it may mediate the phenotypic antibiotic resistance observed on SCFM2 (Fig. 1A and B). However, deletion of the *mtrD* subunit only partially reduced SCFM2-induced resistance to azithromycin (Fig. S1A and B), as well as amoxicillin and ceftriaxone (Fig. S1C and D), indicating that the Mtr pump contributes to but does not fully explain resistance under sputum-like conditions.

To identify broader functional patterns among genes depleted in SCFM2, we performed a COG (clusters of orthologous groups) enrichment analysis (79) on the 46 total and 34 SCFM2-specific depleted gene sets. Of the 21 COG categories analyzed, 5 were significantly over-represented relative to their genome-wide distribution (*P* < 0.05) (Fig. 2E). These included “cell wall/membrane/envelope biogenesis,” encompassing the TAM complex subunit *tamA*, peptidoglycan transpeptidase *mrdA*, and Mtr efflux pump subunits, as well as four metabolic categories: “carbohydrate transport and metabolism,” “amino acid transport and metabolism,” “nucleotide transport and metabolism,” and “coenzyme transport and metabolism.”

Together, these findings indicate that metabolic flexibility and maintenance of envelope integrity are critical for *N. mucosa* replication under sputum-like conditions. These processes may represent key vulnerabilities for therapeutic exploitation in the context of chronic airway colonization.

### Gluconeogenesis and L-lactate catabolism promote *N. mucosa* fitness in synthetic sputum

**Fig 3:**
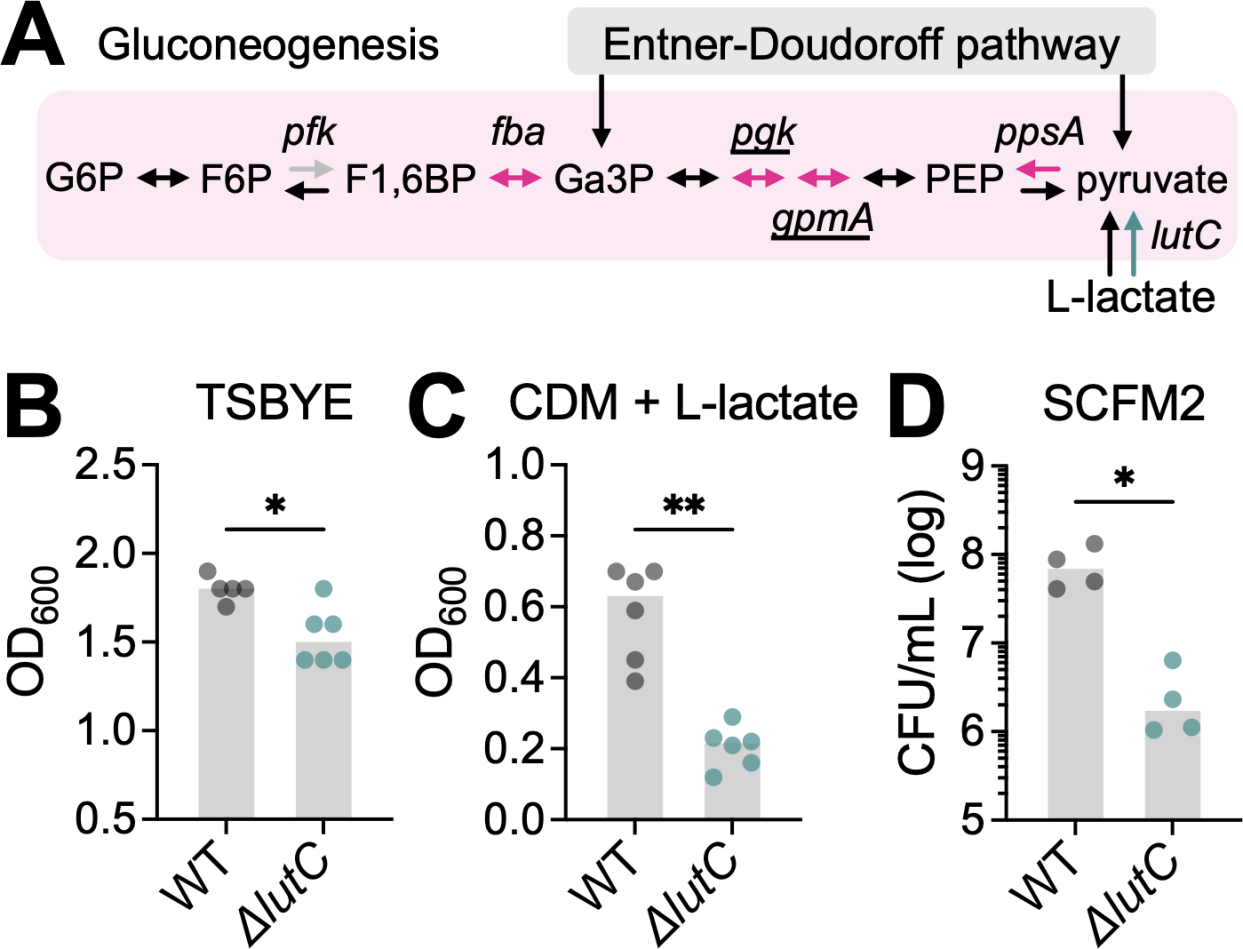
Gluconeogenesis and L-lactate catabolism promote *N. mucosa* fitness in synthetic sputum. (**A**) *N. mucosa* carbon utilization pathways. Enzymes (arrows) highlighted in pink and teal were identified by Tn-seq as fitness determinants in SCFM2. Genes: *pfk*, phosphofructokinase (not present in *N. mucosa* genome); *fba*, fructose 1,6-bisphosphate (F1,6BP) aldolase; *pgk*, phosphoglycerate kinase; *gpmA*, phosphoglycerate mutase; *ppsA*, phosphoenolpyruvate (PEP) synthase; *lutC*, L-lactate dehydrogenase. Underlined genes were also identified as fitness determinants in tryptic soy broth + yeast extract (Fig. 2D). Metabolites: G6P, glucose 6-phosphate; F6P, fructose 6-phosphate; Ga3P, glyceraldehyde 3-phosphate. (**B–D**) Growth yields of the *N. mucosa* wild type (WT) and L-lactate dehydrogenase mutant (*ΔlutC*) after culture for 24 h in (**B**) TSBYE under oxic conditions, (**C**) Socransky’s CDM + 20 mM sodium L-lactate under oxic conditions, or (**D**) SCFM2 under anoxic conditions. Media were not de-oxygenated prior to inoculation at OD_600_ = 0.001 (~10^5^ CFU/mL). CFU were necessary to assess growth in SCFM2 due to its turbidity. Data represent ≥2 biological replicates (performed on separate days), each with ≥2 technical replicates (total *n* = 4–6); gray bars, median; *, *P* < 0.05; **, *P* < 0.01 (two-tailed Mann-Whitney test).

The enrichment of the COG category “carbohydrate transport and metabolism” was driven by four genes involved in glucose metabolism: *fba* (fructose 1,6-bisphosphate aldolase), *pgk* (phosphoglycerate kinase), *gpmA* (phosphoglycerate mutase), and *ppsA* (phosphoenolpyruvate synthase) (Fig. 2B, pink points). As in other *Neisseria* species, *N. mucosa* likely catabolizes glucose via the Entner-Doudoroff (ED) pathway, as it lacks phosphofructokinase (Pfk), a key enzyme in the Embden-Meyerhof-Parnas (EMP) pathway (40) (Fig. 3A). In the ED pathway, glucose is first converted to pyruvate and glyceraldehyde 3-phosphate (Ga3P), which is then further metabolized into pyruvate via enzymes shared with the lower part of the EMP pathway (80) (Fig. 3A). Under glucose-limited conditions, much of the EMP pathway, including Fba, Pgk, and GpmA, can operate in reverse to support gluconeogenesis, regenerating Ga3P and upstream intermediates for anabolic processes.

In many respiratory pathogens, glucose can serve as a key carbon source in artificial sputum (81, 82). In contrast, despite the presence of 3 mM glucose in SCFM2, *N. mucosa* appears to rely more heavily on gluconeogenesis than the ED pathway for fitness in this environment. Supporting this, none of the enzymes specific to the ED pathway were identified as fitness determinants. However, both phosphoenolpyruvate synthase (PpsA), a strictly gluconeogenic enzyme, and fructose-1,6-bisphosphate aldolase (Fba), a reversible enzyme upstream of Ga3P that does not overlap with the ED pathway, were essential for *N. mucosa* fitness in SCFM2 (Fig. 3A).

L-lactate is a gluconeogenic substrate and key *in vivo* carbon source for pathogenic *Neisseria* (83, 84). Like its pathogenic relatives, *N. mucosa* encodes at least two membrane-bound, respiratory L-lactate dehydrogenases: LldD and LutACB (85). Both enzymes oxidize L-lactate to pyruvate while coupling this reaction to cellular respiration, contributing to ATP synthesis via oxidative phosphorylation. Despite the apparent redundancy of these enzymes, only LutACB (specifically LutC) was identified by Tn-seq as a fitness determinant in SCFM2 (Fig. 2B, teal point).

To validate these findings, we constructed a *lutC* deletion mutant. In nutrient-rich TSBYE, the mutant displayed only a mild growth defect (1.2-fold reduction in final yield) compared to the wild type (WT) (Fig. 3B). As expected, this defect was more pronounced (2.9-fold reduction) in a chemically defined medium with L-lactate as the sole carbon source (Fig. 3C). Most strikingly, the *ΔlutC* mutant exhibited a 40-fold reduction in yield relative to the WT in SCFM2 (Fig. 3D), providing strong evidence that L-lactate is a major carbon source for *N. mucosa* under sputum-like conditions.

Given that L-lactate is a gluconeogenic substrate, these findings support the following model for *N. mucosa* carbon utilization in SCFM2: (i) the abundant L-lactate present in this environment (9 mM) is oxidized to pyruvate via LutACB; (ii) this pyruvate is then utilized via gluconeogenesis to generate biosynthetic precursors and support growth (Fig. 3A).

### Amino acid and pyrimidine biosynthesis support *N. mucosa* fitness in synthetic sputum

We next examined the COG category “amino acid transport and metabolism” (Fig. 2C), which was enriched primarily due to six genes involved in the biosynthesis of distinct amino acids or amino acid families: *dapF* (lysine), *proC* (proline), *thrC* (threonine), *aspA* (aspartate), *hisG* (histidine), and *ilvB* (branched-chain amino acids [BCAA] isoleucine, leucine, and valine) (Fig. 2B, purple points).

**Fig 4:**
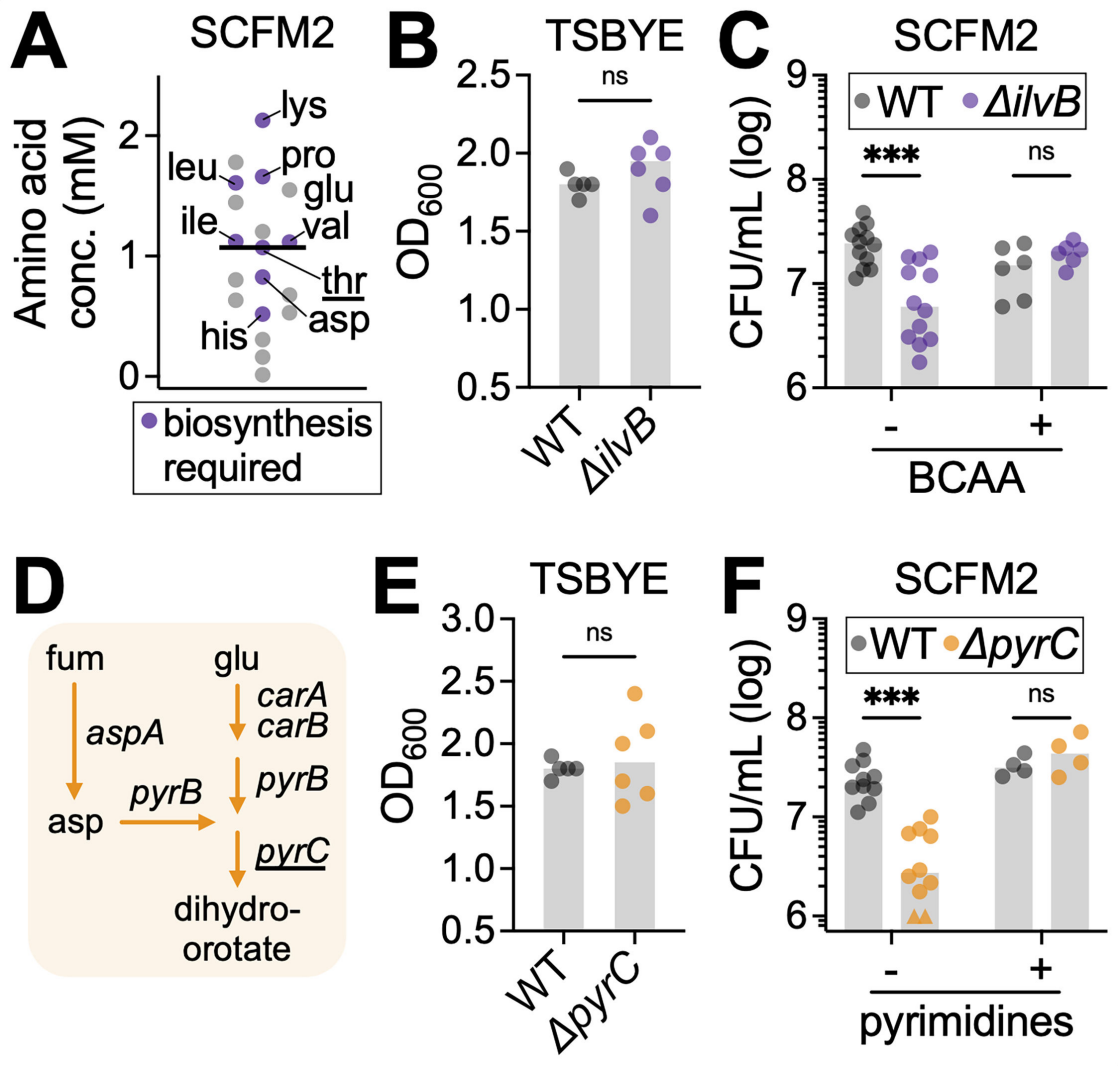
Amino acid and pyrimidine biosynthesis support *N. mucosa* fitness in synthetic sputum. (**A**) Concentration of amino acids in SCFM2. Purple indicates at least one biosynthetic gene was identified by Tn-seq as required for *N. mucosa* fitness in SCFM2. Abbreviations: lys, lysine; pro, proline; glu, glutamate; ile, isoleucine; leu, leucine; val, valine; thr, threonine; asp, aspartate; his, histidine. The biosynthetic gene for threonine (underlined) was also identified as a fitness determinant in tryptic soy broth + yeast extract (Fig. 2D). (**B, C, E, and F**) Growth yields of the *N. mucosa* wild type, acetolactate synthase mutant (*ΔilvB*), and dihydroorotase mutant (*ΔpyrC*) after culture for 24 h in (**B, E**) TSBYE under oxic conditions or (**C, F**) SCFM2 under anoxic conditions. Where indicated, SCFM2 was supplemented with the branched-chain amino acids isoleucine, leucine, and valine (each 10 mM); the pyrimidines cytosine, thymine, and uracil (each 0.2 mM); or an equal volume of H_2_O (vehicle). Media were de-oxygenated in (**C**) and (**F**), but not (**B**) or (**E**), prior to inoculation at OD_600_ = 0.001 (~10^5^ CFU/mL). CFU were necessary to assess growth in SCFM2 due to its turbidity. Data represent ≥2 biological replicates (performed on separate days), each with ≥2 technical replicates (total *n* = 4–12); gray bars, median; ***, *P* < 0.001; ns, not significant (two-tailed Mann-Whitney test). (**F**) For visual clarity, two below-axis values (triangles) were adjusted to 10^6^ CFU/mL. (**D**) *N. mucosa* biosynthetic pathways for the pyrimidine precursor dihydroorotate. All depicted enzymes (arrows) were identified by Tn-seq as fitness determinants in SCFM2. Genes: *carA* and *B*, carbamoyl phosphate synthase small and large subunits; *pyrB*, aspartate carbamoyltransferase; *pyrC*, dihydroorotase; *aspA*, aspartate ammonia-lyase. Metabolites: fum, fumarate; asp, aspartate; glu, glutamate. *pyrC* (underlined) was also identified as a fitness determinant in TSBYE (Fig. 2D).

Surprisingly, all eight amino acids associated with these genes are present in SCFM2, with six of the eight exceeding the median amino acid concentration (Fig. 4A). This suggests that, despite their presence, amino acid levels in SCFM2 are not sufficient to meet *N. mucosa*’s metabolic demands, necessitating *de novo* synthesis. Notably, *dapF* contributes not only to lysine biosynthesis but also to the production of meso-2,6-diaminopimelate, a key precursor for peptidoglycan (76). Its essentiality may therefore reflect dual roles in supporting both amino acid and cell wall biosynthesis, consistent with the enrichment of the “cell wall/membrane/envelope biogenesis” COG category (Fig. 2C).

To validate the role of amino acid biosynthesis, we constructed a deletion mutant of *ilvB*, which encodes a subunit of acetolactate synthase, the first committed step in BCAA biosynthesis (86). While this mutant grew comparably to the WT in nutrient-rich TSBYE (Fig. 4B), it exhibited a fourfold reduction in yield in SCFM2, consistent with Tn-seq results (Fig. 4C). Supplementing SCFM2 with exogenous BCAA fully restored the mutant’s growth to WT levels, confirming that limited BCAA availability constrains *N. mucosa* fitness in this environment (Fig. 4C).

The parallel enrichment of the COG category “nucleotide transport and metabolism” (Fig. 2C) was driven primarily by four genes involved in pyrimidine biosynthesis: *carA*, *carB*, *pyrB*, and *pyrC* (Fig. 2B, orange points). This pathway uses L-glutamate and L-aspartate, both present in SCFM2 (Fig. 4A, labeled “glu” and “asp”), to synthesize dihydroorotate, a central intermediate in pyrimidine biosynthesis (87) (Fig. 4D). In addition, *aspA*, which synthesizes L-aspartate from fumarate, was also required, linking pyrimidine biosynthesis to central carbon metabolism (Fig. 4D).

Although SCFM2 contains DNA as a structural component, it lacks free pyrimidines (cytosine, thymine, and uracil), likely explaining *N. mucosa*’s strong reliance on *de novo* synthesis. To validate this, we deleted *pyrC*, which encodes dihydroorotase (Fig. 4D). As with the *ΔilvB* mutant, the *ΔpyrC* mutant grew normally in TSBYE (Fig. 4E) but exhibited an eightfold reduction in SCFM2 (Fig. 4F). This defect was fully rescued by exogenous pyrimidines, confirming that pyrimidine limitation restricts *N. mucosa* growth under sputum-like conditions (Fig. 4F).

Together, these findings highlight the nutritional constraints that are imposed on *N. mucosa* in SCFM2. Despite the presence of amino acids and nucleotides, their concentrations—or bioavailable forms—are insufficient to support optimal growth, driving *N. mucosa* to perform *de novo* synthesis to meet its metabolic needs.

### Nitrate respiration promotes *N. mucosa* fitness in synthetic sputum

**Fig 5:**
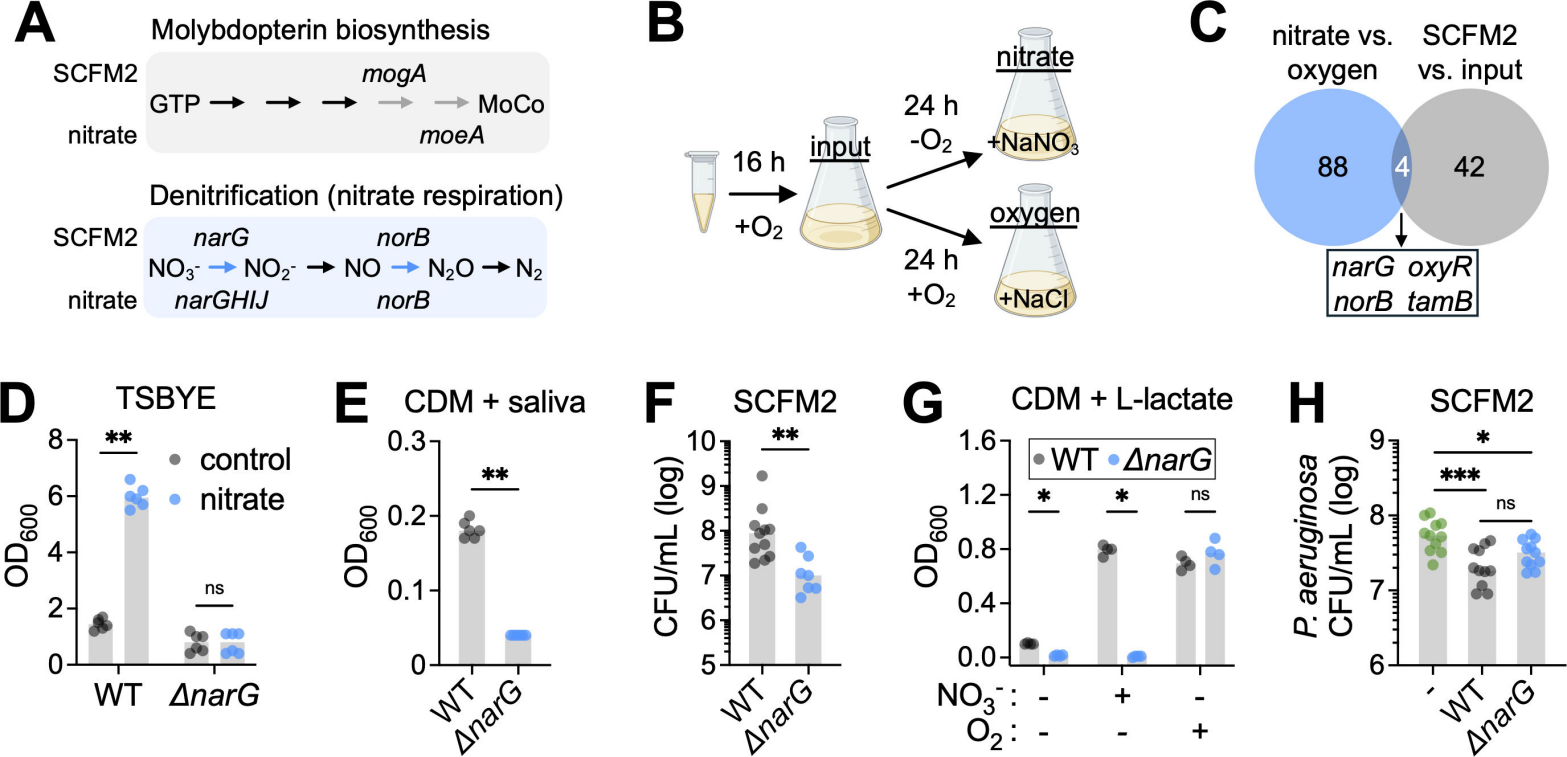
Nitrate respiration promotes *N. mucosa* fitness in synthetic sputum. (**A**) *N. mucosa* molybdopterin biosynthesis and denitrification pathways. Enzymes (arrows) highlighted in gray and blue were identified by Tn-seq as fitness determinants in SCFM2 or nitrate-supplemented tryptic soy broth + yeast extract. Gene names above arrows: significant in SCFM2. Gene names below arrows: significant in nitrate-supplemented TSBYE. Genes: *mogA*, molybdopterin adenylyltransferase; *moeA*, molybdopterin molybdenum transferase; *narG*, nitrate reductase alpha (catalytic) subunit; *narHIJ*, nitrate reductase beta subunit, gamma subunit, and chaperone protein; *norB*, nitric oxide reductase catalytic subunit. Metabolites: GTP, guanosine triphosphate; MoCo, molybdenum cofactor; NO_3_^−^, nitrate; NO_2_^−^, nitrite; NO, nitric oxide; N_2_O, nitrous oxide; N_2_, nitrogen gas. Tn-seq results for *moeA*, *narH*, *narI*, and *narJ* were marginally below the fold change cutoff for significance (*P*_adj_ <0.03, but FC only 1.8–2). (**B**) Transposon-seq experimental design. A cryo-aliquot of the *N. mucosa* Tn mutant pool was revived for 16–20 h in TSBYE under oxic conditions (input condition), then back-diluted and cultured for 24 h in TSBYE + 10 mM NaNO_3_ under anoxic conditions (nitrate condition) or TSBYE + 10 mM NaCl under oxic conditions (oxygen condition). Media were de-oxygenated prior to inoculation. (**C**) Venn diagram showing the overlap between the number of *N. mucosa* fitness determinants in TSBYE + nitrate (when compared to TSBYE + oxygen) and SCFM2 (when compared to the input). (**D–G**) Growth yields of the *N. mucosa* wild type and nitrate reductase mutant (*ΔnarG*) after culture for 24 h in (**D**) TSBYE + 10 mM NaCl (control) or NaNO_3_ (nitrate) under anoxic conditions, (**E**) Socransky’s chemically defined medium + 25% saliva (pooled from healthy human donors) under anoxic conditions, (**F**) SCFM2 under anoxic conditions, or (**G**) CDM + 20 mM sodium L-lactate under the indicated conditions (−O_2_, anoxic; +O_2_, oxic; −NO_3_^−^, +10 mM NaCl; +NO_3_^−^, +10 mM NaNO_3_). Media were not de-oxygenated prior to inoculation at OD_600_ = 0.001 (~10^5^ CFU/mL). CFU were necessary to assess growth in SCFM2 due to its turbidity. (**F**) The WT data are the same as the SCFM2 data in Fig. 1C. (**H**) Growth yields of *P. aeruginosa* PA14 after culture for 24 h in SCFM2 under anoxic conditions in mono- or co-culture with the *N. mucosa* WT or *ΔnarG* mutant. Media were de-oxygenated prior to inoculation with ~10^3^ CFU/mL for *P. aeruginosa* and ~10^5^ CFU/mL for *N. mucosa*. (**D–H**) Data represent ≥2 biological replicates (performed on separate days), each with 1–4 technical replicates (total *n* = 4–11); gray bars, median; *, *P* < 0.05; **, *P* < 0.01; ***, *P* < 0.001; ns, not significant (two-tailed Mann-Whitney test).

The near-significant enrichment of the COG category “coenzyme transport and metabolism” (*P* = 0.06) was primarily driven by genes involved in the biosynthesis of thiamine (*thiE*), glutathione (*gshA*), and molybdopterin (*mogA*) (Fig. 2C). Of these, molybdopterin was of particular interest, as it is an essential cofactor for anaerobic reductases (60) (Fig. 5A). These enzymes enable respiration using alternative terminal electron acceptors under anoxic conditions, suggesting that anaerobic respiration may contribute to *N. mucosa*’s enhanced growth in SCFM2 under oxygen-limited conditions (Fig. 1C). Supporting this, *N. mucosa* encodes genes for denitrification, a respiratory pathway that reduces nitrate (NO_3_^-−^) to nitrogen gas (N_2_) (88), and two such genes, *narG* (nitrate reductase) and *norB* (nitric oxide reductase), were identified as fitness determinants in SCFM2 (Fig. 5A).

To more directly assess genes required for growth with nitrate, we performed additional Tn-seq experiments under defined conditions. Specifically, we compared growth in nutrient-rich TSBYE under anoxic conditions with nitrate as the terminal electron acceptor vs growth under oxic conditions with oxygen as the acceptor (Fig. 5B). This analysis identified 92 genes that are specifically required for nitrate-supported anaerobic growth but dispensable in the presence of oxygen (Fig. 5C).

Of these 92 genes, 4 overlapped with SCFM2 fitness determinants, representing a trend toward significant enrichment (*P* = 0.056, one-sided hypergeometric test) (Fig. 5C). As expected, two of these genes, *narG* and *norB*, encode core components of the denitrification pathway (Fig. 5A). A third, *oxyR*, encodes a transcriptional regulator involved in oxidative and nitrosative stress responses, potentially related to nitric oxide-induced toxicity during denitrification (89). The fourth gene, *tamB*, encodes a subunit of the translocation and assembly module complex required for outer membrane protein assembly (75). Notably, *tamB* exhibited the most pronounced depletion among all SCFM2 fitness determinants (log_2_ fold change <−22; Fig. 2B), further linking nitrate respiration to broader fitness requirements under sputum-like conditions.

To validate the role of nitrate respiration, we constructed a *narG* deletion mutant. Unlike the WT, the *ΔnarG* mutant failed to achieve enhanced growth in TSBYE supplemented with nitrate (Fig. 5D). The mutant was also attenuated in a chemically defined medium supplemented with pooled human saliva, a physiologically relevant nutrient source in the oral cavity, where nitrate can temporarily reach levels as high as 5–10 mM following nitrate-rich meals (90) (Fig. 5E). Most notably, the *ΔnarG* mutant exhibited a ninefold reduction in yield compared to the WT in SCFM2, consistent with Tn-seq results (Fig. 5F).

Given that L-lactate is a key non-fermentable carbon source for *N. mucosa* in SCFM2 (Fig. 3), we next tested whether nitrate respiration enables L-lactate utilization under anoxic conditions. Indeed, the *ΔnarG* mutant showed a 7-fold reduction in anoxic growth yield in CDM supplemented with L-lactate, which increased to an 80-fold defect with added nitrate (10 mM; Fig. 5G). These defects were specific to anaerobic metabolism, as the mutant grew comparably to the WT under oxic conditions (Fig. 5G).

In the inflamed airway, *N. mucosa* likely faces competition from other nitrate-respiring bacteria, particularly *Pseudomonas aeruginosa*, which also depends on nitrate respiration for fitness in synthetic sputum (58). To assess the impact of nitrate respiration on interspecies competition, we compared *P. aeruginosa* growth in mono- and co-culture with either the *N. mucosa* WT or *ΔnarG* mutant in SCFM2 (Fig. 5H). Co-culture with the WT significantly reduced *P. aeruginosa* yield (2.9-fold), whereas co-culture with the *ΔnarG* mutant had a more modest effect (1.7-fold), suggesting that nitrate respiration contributes to *N. mucosa*’s competitive fitness in polymicrobial settings (Fig. 5H).

Together, these results demonstrate that nitrate respiration is a key fitness determinant for *N. mucosa* under sputum-like conditions. It not only supports anoxic growth on L-lactate but also enhances competitive interactions with other nitrate-utilizing airway pathogens.

### Targeting *N. mucosa* metabolic vulnerabilities in synthetic sputum

Having identified key genes required for *N. mucosa* fitness in SCFM2, we next explored whether any of these metabolic functions could be selectively disrupted using non-antibiotic compounds. A major motivation was our observation that *N. mucosa* displays reduced susceptibility to antibiotics under sputum-like conditions (Fig. 1A and B), potentially contributing to the limited efficacy of conventional antibiotic therapies.

We first examined *N. mucosa*’s dependence on L-lactate catabolism via the L-lactate dehydrogenase complex LutACB, which was a strong fitness determinant in SCFM2 (Fig. 3D). Treatment with oxalate, a known inhibitor of L-lactate dehydrogenases (91), impaired *N. mucosa* growth in SCFM2 but not in nutrient-rich TSBYE (Fig. S2A and B), consistent with the differential requirement for *lutC* in these environments (Fig. 3B and D). However, in competition experiments where the WT and *ΔlutC* strains were co-inoculated at an initial 1:1 ratio, oxalate treatment failed to selectively inhibit the WT, which still robustly outcompeted the *ΔlutC* mutant even in the presence of oxalate (Fig. S2C). This result suggests that oxalate exerts broader, non-selective inhibitory effects in SCFM2, reducing its utility for targeting L-lactate metabolism.

We next tested whether inhibition of *de novo* pyrimidine biosynthesis could selectively impair *N. mucosa* in SCFM2. We focused on the small molecule PALA (N-phosphonacetyl-L-aspartate), an inhibitor of aspartate carbamoyltransferase (*pyrB*), which was required for fitness in SCFM2 (Fig. 4D). Consistent with PALA’s reported context-dependent activity (92), PALA inhibited *N. mucosa* growth in SCFM2 but not TSBYE (Fig. S2D and E). However, as with oxalate, PALA failed to selectively inhibit the WT. In competition assays, the *ΔpyrC* mutant (which should be resistant to PALA due to its downstream position in the pathway) was outcompeted by the WT even under PALA treatment (Fig. S2F), suggesting PALA may also have off-target or general inhibitory effects in this context.

**Fig 6:**
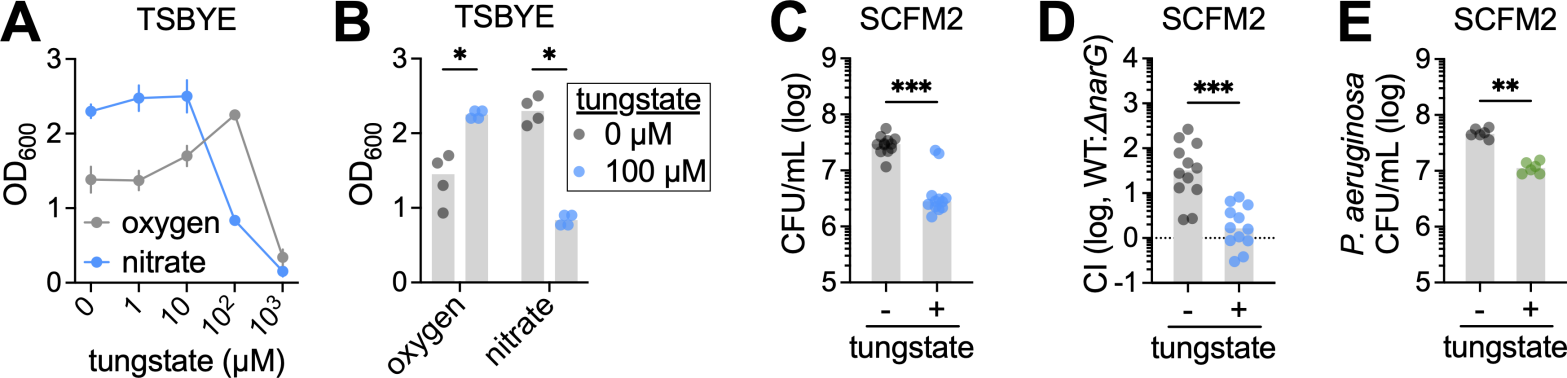
Tungstate selectively inhibits *N. mucosa* nitrate respiration in synthetic sputum. (**A and B**) Growth yields of *N. mucosa* after culture for 24 h in tryptic soy broth + yeast extract with increasing amounts of tungstate. Oxygen: TSBYE + 10 mM NaCl incubated under oxic conditions. Nitrate: TSBYE + 10 mM NaNO_3_ incubated under anoxic conditions. For visual clarity, statistical comparisons are only shown in (**B**) for 0 and 100 µM sodium tungstate. (**C and E**) Growth yields of (**C**) *N. mucosa* and (**E**) *P. aeruginosa* PA14 after culture for 24 h under anoxic conditions in SCFM2 + 100 µM sodium tungstate or an equal volume of H_2_O (-). (**D**) Competitive indexes (CI) for the *N. mucosa* wild type and nitrate reductase mutant (*ΔnarG*) after co-culture for 24 h under anoxic conditions in SCFM2 + 100 µM sodium tungstate or an equal volume of H_2_O (-). CI determined by dividing the output WT:mutant ratio by the input WT:mutant ratio (~1:1); a CI of 1 (log_10_CI = 0; dotted line) indicates equal fitness between WT and mutant. (**A–E**) Media were de-oxygenated in (**C–E**), but not (**A**) or (**B**), prior to inoculation at OD_600_ = 0.001 (~10^5^ total CFU/mL). CFU were necessary to assess growth in SCFM2 due to its turbidity. Data represent ≥2 biological replicates (performed on separate days), each with ≥2 technical replicates (total *n* = 4–12); (**A**) points, mean ± standard error; (**B–E**) gray bars, median; *, *P* < 0.05; **, *P* < 0.01; ***, *P* < 0.001 (two-tailed Mann-Whitney test).

Lastly, we investigated whether nitrate respiration, another essential function in SCFM2 (Fig. 5F), could be disrupted using tungstate (WO_4_^2−^), an analog of molybdate (MO_4_^2−^) that inhibits molybdo-enzymes such as nitrate reductase (60). In TSBYE, tungstate at 100 µM impaired growth under nitrate-respiring conditions while enhancing growth under aerobic conditions, indicating selective inhibition of nitrate reductase (Fig. 6A and B). At higher concentrations (1 mM), tungstate impaired growth under both conditions, likely due to broader metabolic disruption (Fig. 6A). Based on this, 100 µM tungstate was used for subsequent assays. In SCFM2, this concentration strongly suppressed *N. mucosa* growth under anoxic conditions (Fig. 6C) and critically abolished the competitive advantage of the WT over the *ΔnarG* mutant (Fig. 6D). To determine whether this strategy might extend to other airway pathogens, we tested tungstate against *P. aeruginosa*. As observed with *N. mucosa*, *P. aeruginosa* growth in SCFM2 was significantly impaired by tungstate (Fig. 6E).

Together, these findings underscore the importance of studying microbial physiology in disease-relevant environments. Metabolic pathways that are dispensable in nutrient-rich media may be critical for survival in the inflamed airway, revealing context-specific vulnerabilities not apparent under standard laboratory conditions. Targeting such conditional dependencies offers a promising strategy to suppress opportunistic pathogens such as *N. mucosa*, particularly in settings where conventional antibiotics are less effective.

## DISCUSSION

In this study, we identified key metabolic pathways that support the fitness of *N. mucosa* in synthetic cystic fibrosis medium, a validated *in vitro* model that mimics the nutrient environment of the chronically inflamed lower airway (51–55). Our results demonstrate that L-lactate catabolism, *de novo* amino acid and pyrimidine biosynthesis, and anaerobic nitrate respiration are essential for *N. mucosa* growth and survival under sputum-like conditions. These findings provide new mechanistic insight into how oral commensals metabolically adapt to disease-associated microenvironments (17–20).

*N. mucosa* and related commensal *Neisseria* species are typically regarded as health-associated taxa, predominating in the upper airway microbiota of healthy individuals, including the tongue and dental plaque (14, 36). However, growing evidence implicates these organisms in chronic lung diseases such as bronchiectasis, cystic fibrosis, and COPD (12, 13, 21–23, 37). Our data support this emerging view by suggesting that *N. mucosa* can engage metabolic programs, akin to those of pathogenic *Neisseria* (39, 40), to persist in the inflamed airway.

Specifically, we found that *N. mucosa* proliferates in SCFM2 under anoxic conditions by utilizing two inflammation-associated metabolites: (i) L-lactate, a non-fermentable carbon source, and (ii) nitrate, an anaerobic electron acceptor (44–47). Pathogenic *Neisseria*, such as *N. gonorrhoeae*, similarly depend on L-lactate catabolism for successful host colonization (83, 84), likely exploiting its accumulation during inflammation due to heightened glycolysis in activated immune and epithelial cells (44, 45, 93). Although pathogenic *Neisseria* are unable to respire nitrate (NO_3_^−^), they can survive under anoxic conditions by respiring nitrite (NO_2_^−^) (72, 88), a major byproduct (alongside nitrate) of inducible nitric oxide synthase (iNOS) activity (46, 57, 59). While critical for host defense, the upregulation of iNOS during airway inflammation likely creates a niche for nitrate-respiring taxa by increasing local nitrate and nitrite availability (46, 47). These parallels suggest that pathogenic and commensal *Neisseria* capitalize on inflammation-derived metabolites to support growth in oxygen-limited environments.

While lactate and nitrate metabolism may promote *N. mucosa* invasion of the inflamed lower airway, these pathways are most likely maintained because they support the organism’s commensal lifestyle. For instance, we found that nitrate respiration is required for *N. mucosa* to subsist on human saliva, a major nutrient source in the oral cavity (94). This aligns with the known accumulation of dietary nitrate to high (millimolar) levels within saliva, as well as the widespread capacity for nitrate respiration among oral commensals (90, 95, 96). Additionally, lactate is a major fermentation product of commensal oral streptococci, which “cross-feed” lactate to neighboring lactate-utilizing taxa, fostering physical co-association between producers and consumers (15, 91). Thus, the ability to utilize lactate and nitrate may have evolved primarily to support *N. mucosa* colonization of the healthy upper airway but is co-opted to support its survival in the inflamed lower airway.

This metabolic conservation underscores a key point: while pathogens likely retain nitrate and lactate utilization as adaptations to inflammation, commensals like *N. mucosa* may have developed these functions in the absence of inflammation but benefit opportunistically when such metabolites are abundant during disease. Indeed, other oral commensals such as *Veillonella*, which also metabolize lactate and nitrate, have been observed to translocate to distal inflamed sites (e.g., the gut of patients with inflammatory bowel disease), raising the possibility that inflammation inadvertently selects for organisms pre-adapted to health-associated metabolic niches (7, 97, 98). In the lung, oral commensals likely reach the lower airways via micro-aspiration, where the metabolic overlap between health and disease contexts facilitates their expansion in chronic infection.

Beyond microbe-host interactions, our results point toward microbe-microbe competition as another critical factor shaping airway colonization. As noted above, nitrate utilization is not unique to *Neisseria*; it is also widespread among other oral taxa, such as *Rothia* species, which co-expand alongside *Neisseria* in response to dietary nitrate and are frequent inhabitants of the cystic fibrosis lung (96, 99). The presence of multiple nitrate-respiring commensals in this niche illustrates how nutrient availability, particularly of alternative electron acceptors like nitrate, can shape microbial community structure (100). As a result, *N. mucosa* is likely to experience niche overlap not only with canonical bronchiectasis pathogens like *P. aeruginosa* but also with fellow nitrate-respiring oral commensals, all competing within the same resource-limited airway environment.

Our study also highlights “phenotypic,” or environmentally induced, antibiotic resistance (65), a phenomenon previously observed among other respiratory pathogens under sputum-like conditions (101, 102). Specifically, we found that *N. mucosa* exhibits increased resistance to multiple, clinically relevant antibiotics when cultured on SCFM2. While the underlying mechanism remains unclear, our data suggest that the Mtr efflux pump may contribute to this response. Further work is needed to elucidate the complete mechanisms, as well as to identify the specific sputum-derived factor(s) that trigger this resistance. Additional Tn-seq experiments, particularly those involving *N. mucosa* cultured in SCFM2 and exposed to antibiotics, could provide valuable insight into these questions.

Given the limitations of conventional antibiotics (50, 63, 64), our findings provide proof of concept that targeting infection-specific metabolic pathways may be a promising avenue for the discovery of alternative therapeutics, particularly those overlooked in traditional drug screens using nutrient-rich media. For example, building on pioneering work in the gut microbiome field (60, 103), we found that nitrate respiration—a pathway required for *N. mucosa* fitness in SCFM2 and saliva—can be selectively inhibited using tungstate, a molybdo-enzyme antagonist. While tungstate’s use in the clinic may be limited by potential toxicity (104), its effectiveness against both *N. mucosa* and *P. aeruginosa* in disease-relevant media supports the broader therapeutic potential of targeting nitrate respiration or other inflammation-associated metabolic dependencies. This approach could be relevant not only for chronic diseases of the lower airway but also at other barrier sites, such as the skin, vaginal tract, or oral cavity.

In conclusion, our study reveals that *N. mucosa* leverages a set of inflammation-compatible metabolic strategies, likely evolved for life in the healthy oral cavity, to persist in the inflamed lower airway. By uncovering the physiological requirements that enable this opportunistic shift, we lay the groundwork for the development of novel, non-antibiotic therapeutics that target context-specific metabolic vulnerabilities at the site of infection, with potential application across a range of chronic inflammatory diseases.

## MATERIALS AND METHODS

### Strains, media, and growth conditions

*Neisseria mucosa* ATCC 19696 and *Pseudomonas aeruginosa* PA14 were used as wild-type strains. Both were routinely cultured in tryptic soy broth + 0.5% (wt/vol) yeast extract, or on solid TSBYE + 1.5% (wt/vol) agar. Synthetic cystic fibrosis medium (51) was purchased from SynthBiome (#10002, version not zinc limited). SCFM2 was aliquoted and stored at −20°C until use. A modified version of Socransky’s chemically defined medium (105) was prepared as described (106), with CaCl_2_ omitted. Pooled saliva (15–20 mL) was collected from three to four healthy human donors, centrifuged to remove debris (~3,200 × *g*, 5–10 min), filter sterilized (0.2 µm pore size), aliquoted, and stored at −20°C until use. Oxic cultures were incubated in 5% CO_2_. Anoxic cultures were incubated in an anaerobic chamber (Coy Laboratory Products or Don Whitley Scientific) containing 90% N_2_, 5% CO_2_, and 5% H_2_. Both oxic and anoxic liquid cultures were incubated without shaking. For some anoxic assays, media and polystyrene culture tubes were pre-reduced overnight in either an anaerobic chamber or, for SCFM2, at 4°C in anaerobic jars (using sachets that generate anoxic conditions). PALA (NSC-224131) (107) was a generous gift from Dr. Christine McDonald (Cleveland Clinic).

### Growth assays

Colonies from streak plates were inoculated into TSBYE and incubated overnight (16–24 h) under oxic conditions. Cultures were pelleted (4,000 × *g*, 1–2 min), washed with PBS, and adjusted to OD_600_ = 1.0 (or 0.5) in PBS. Cells were then diluted 1:1,000 (or 1:500) into test media to initial OD_600_ = 0.001 (~10^5^ CFU/mL). Cultures were incubated for ~24 h under oxic or anoxic conditions before assessing growth, either by measuring OD_600_ or determining CFU (the latter being necessary for SCFM2 due to its high turbidity). For competition assays, WT and mutant strains were both adjusted to OD_600_ = 1.0 (or 0.5), mixed at a 1:1 ratio, and diluted 1:1,000 (or 1:500) into test media. For co-culture with *P. aeruginosa*, *N. mucosa* was adjusted to OD_600_ = 0.5 and *P. aeruginosa* to OD_600_ = 0.005 prior to 1:500 dilution into test media. Growth assays were conducted in 1 mL volumes using 4 mL polystyrene tubes (12 × 75 mm). Where indicated in figure legends, test media were de-oxygenated prior to inoculation. De-oxygenation was not performed routinely until after it was found to be critical for experiments involving tungstate.

### CFU determination

CFU were determined as described (108). Briefly, cultures were serially diluted in PBS in 96-well plates (180 µL/well), and using a multichannel pipette, 5 µL of each 10-fold dilution was spotted five times onto TSBYE agar (one plate per sample, 25 µL total per dilution). To determine CFU for *N. mucosa* mutants in competition assays, serial dilutions were spotted onto TSBYE agar + 40 µg/mL kanamycin, while to determine CFU for *P. aeruginosa*, dilutions were spotted onto *Pseudomonas* isolation agar.

### MIC assays

Minimum inhibitory concentrations were determined using MIC test strips (Liofilchem) on 0.5× TSBYE and SCFM2 agar. Half-strength agar was prepared by mixing 1× liquid media (either 1× TSBYE + 20 mM NaCl or 1× SCFM2, warmed to 37°C) with an equal volume of autoclaved 2× agar (3% [wt/vol], cooled to 55°C). Lawns were formed by spreading PBS-washed overnight cultures (OD_600_ = 1.0) onto plates using cotton swabs. After a 4-h pre-incubation under oxic conditions (to allow induction of resistance mechanisms), MIC strips were applied, and plates incubated for an additional ~20 h under oxic conditions. Anoxic MICs were attempted but not reported due to poor lawn formation, even on 0.5× TSBYE agar + 10 mM NaNO_3_.

### *N. mucosa* Tn mutant pool

The *N. mucosa* transposon mutant pool was generated by adapting a previously described method for *Acinetobacter baylyi* (109). In this approach, genomic DNA is mutagenized *in vitro* using EZ-Tn5 transposase (Biosearch Technologies) and then introduced into the target organism via natural transformation. The EZ-Tn5 transposon was generated by PCR-amplifying the kanamycin resistance cassette from plasmid pYGK (110) using 5′-phosphorylated primers containing EZ-Tn5 transposase recognition sequences at their 5′ ends (see Table S1 for sequences). The *in vitro* mutagenesis and gap repair reactions were performed as described in the original protocol but scaled up fivefold: 8  µg of *N. mucosa* genomic DNA was mutagenized in a 150 µL reaction and gap repaired in a 250 µL reaction. Parallel negative control reactions substituted H_2_O for both transposase and transposon.

To perform the natural transformation, a colony of *N. mucosa* was re-struck onto TSBYE agar and incubated overnight under oxic conditions. Cells were harvested from the plate into TSBYE + 5 mM MgCl_2_, adjusted to OD_600_ = 2.0, and mixed 1:1 with the unpurified 250 µL gap repair reaction. The mixture was spotted onto five polycarbonate filters (0.2 µm pore size), placed on TSBYE agar, and incubated for 2.5 h under oxic conditions. Cells were then harvested from the filters by vortexing into ~10  mL TSBYE + 25% glycerol and stored as 2 mL aliquots at −80°C. One aliquot was thawed, diluted in TSBYE, and spread across ~30 different 150 mm TSBYE agar plates + 40  µg/mL kanamycin, followed by incubation for ~36 h under oxic conditions. The resulting transformants (on average, >2,000 colonies per plate) were harvested into ~100  mL TSBYE + 25% glycerol and stored as 1 mL aliquots at −80°C. No transformants were observed for the negative-control gap repair reaction.

### Tn-seq experiments

Tn-seq was performed on the *N. mucosa* Tn mutant pool under four conditions: input, SCFM2, nitrate, and oxygen. Each condition was tested in biological triplicate (on separate days), for a total of 12 samples. At the end of each experiment, cultures were pelleted and stored at −20°C until further processing.

#### Input condition

For each replicate, a frozen aliquot of the *N. mucosa* Tn mutant pool was thawed, and 0.5  mL was inoculated into 50  mL TSBYE in a 250 mL flask. Cultures were incubated under oxic conditions for 17–20 h, corresponding to six to seven generations based on initial vs final CFU/mL.

#### SCFM2 condition

Each replicate was initiated from an input culture as described above. A portion of the culture was pelleted at 4,000  × * g* for 1  min, resuspended in SCFM2, and adjusted to OD_600_ = 1.0. The suspension was then diluted 1:1,000 (initial OD_600_ = 0.001) into 50  mL SCFM2 in a 250 mL flask and incubated under anoxic conditions for 24  h (eight to nine generations based on initial vs final CFU/mL). SCFM2 was not pre-reduced for Tn-seq experiments.

#### Nitrate and oxygen conditions

Each replicate was initiated from an input culture as described above. A portion of the culture was directly diluted to initial OD_600_ = 0.01 (i.e., without intermediate OD adjustment) in 50 mL de-oxygenated TSBYE + 10 mM NaNO_3_ (nitrate condition) or 10 mM NaCl (oxygen condition) in a 250 mL flask, and incubated under anoxic or oxic conditions, respectively, for 24 h (seven to eight generations based on initial vs final OD_600_).

### Tn-seq library preparation

Genomic DNA was isolated from cell pellets as described (111), with minor modifications (specifically, mutanolysin and lyticase were omitted from the enzymatic lysis step). Tn-seq libraries were then prepared using a two-step PCR method, largely as described (112). Briefly, genomic DNA was (i) sheared, (ii) C tailed using terminal deoxynucleotidyl transferase, (iii) PCR amplified for 15 cycles using a 5′-biotinylated Tn-specific forward primer and a C-tail-specific reverse primer (olj376), (iv) purified using streptavidin-coated magnetic beads, and (v) PCR amplified for 20 additional cycles using a nested Tn-specific forward primer and an olj376-specific reverse primer, both containing Illumina adapter sequences at their 5′ ends. Pooled libraries were sequenced on an Illumina NovaSeq (2 × 150 reads) at the Genome Technology Access Center (McDonnell Genome Institute, Washington University in St. Louis).

### Tn-seq analysis

Tn-seq data were analyzed largely as described (112), using only the R1 read from paired-end sequencing. The analysis proceeded in five main steps.

1. Read pre-processing (Cutadapt v4.6): (i) reads were filtered for and trimmed of the 5′ Tn sequence TTCAGATGTGTATAAGAGACAG, (ii) trimmed to remove 3′ C-tails (≥12 consecutive C’s) and low-quality bases (*Q* <20, fastq format), and (iii) truncated to a length of 20–25 bp.
2. Alignment (Bowtie 2 v2.5.3) (113): processed reads were aligned to a re-sequenced assembly of the *N. mucosa* 19696 genome (described below) using default end-to-end settings.
3. Filtering and insertion site analysis (command line): (i) lower-quality alignments (*Q* <40, sam format) were discarded, and (ii) unique Tn insertion sites per sample were identified and counted.
4. Read counting (Rsubread v2.12.3) (114): read counts per gene (i.e., Tn mutant abundance) were quantified using featureCounts() with a saf annotation file derived from the re-sequenced genome.
5. Differential analysis (DESeq2 v1.42.1) (115): a single counts matrix including all 12 samples was used to normalize read counts for library size and to perform differential abundance analysis using the Wald test with Benjamini-Hochberg correction for multiple testing.

A summary of each analysis step is provided in Data set S1, along with the following files: the saf-format annotation file used with Rsubread, the DESeq2-normalized counts matrix, and the DESeq2 result files for the comparisons SCFM2 vs input, nitrate vs input, oxygen vs input, and nitrate vs oxygen.

### *N. mucosa* genome re-sequencing

The *N. mucosa* 19696 genome was re-sequenced to support accurate Tn-seq analysis, as the existing NCBI assembly (ASM302831v1, as of 18 June 2025) was flagged as contaminated. Genomic DNA was submitted to SeqCenter for hybrid Nanopore long-read/Illumina short-read sequencing, *de novo* assembly, and genome annotation. The resulting gff annotation file was modified in Excel to create the saf-format annotation file for Rsubread. KEGG *K*-number annotations were generated by: (i) extracting all gene nucleotide sequences from the re-sequenced genome using the BEDTools getfasta function (116) and (ii) submitting the resulting fasta file to the KEGG Automatic Annotation Server (117) using the following settings, such as BLAST as the search algorithm, the prokaryote representative gene set, and the BBH (bi-directional best hit) assignment method. COG annotations were generated as described (118).

### *N. mucosa* deletion mutants

Deletion mutants were generated by naturally transforming *N. mucosa* with DNA constructs designed to replace the target gene with a kanamycin resistance cassette. Each construct consisted of the kanamycin cassette flanked by ~1 kb regions upstream and downstream of the target gene. To assemble constructs, the flanking regions were PCR amplified from *N. mucosa* 19696 genomic DNA and the kanamycin cassette from plasmid pYGK (110) (see Table S1 for primer sequences). Assembly was performed using the HiFi DNA Assembly Master Mix (New England Biolabs). Primers for the flanking regions were designed using the NCBI *N. mucosa* 19696 genome assembly (ASM302831v1) and included specific overhangs to facilitate both transformation and assembly: (i) the upstream forward and downstream reverse primers included the *Neisseria* DNA uptake sequence at their 5′ ends to enhance natural transformation (119). (ii) The upstream reverse and downstream forward primers included sequences complementary to the ends of the kanamycin cassette to enable DNA assembly. Full-length assembled constructs (~3 kb) were PCR amplified using the upstream forward and downstream reverse primers and were confirmed by agarose gel electrophoresis. For transformations, *N. mucosa* colonies were re-struck onto TSBYE agar and incubated overnight under oxic conditions. Cells were then harvested into TSBYE + 5 mM MgCl_2_, adjusted to OD_600_ = 2.0, and mixed 1:1 with the DNA construct (≥500 ng in 50 µL). The mixture (100 µL total) was spotted onto a polycarbonate filter (0.2 µm pore size), placed on TSBYE agar, and incubated for 2.5 h under oxic conditions. Cells were subsequently harvested into TSBYE and plated on TSBYE agar + 40 µg/mL kanamycin. Transformants were screened by PCR to confirm deletion of the target gene.

### Statistical analysis

Two-tailed Mann-Whitney tests were performed using GraphPad Prism. Wald tests for Tn-seq data were conducted in R using the DESeq2 package with default settings. Hypergeometric tests were performed in Microsoft Excel using the hypgeomdist function.

## Data Availability

Raw Tn-seq and whole-genome sequencing data have been deposited in the NCBI Sequence Read Archive under BioProject accession numbers PRJNA1280941 and PRJNA1280957, respectively.

## SUPPLEMENTAL MATERIAL

Data set S1 mbio.01948-25-s0001.xlsx full legend in separate attachment

Fig. S1 mbio.01948-25-s0002.tiff The Mtr efflux pump partially mediates the enhancement of *N. mucosa* antibiotic resistance on SCFM2.

Fig. S2 mbio.01948-25-s0003.tiff Inhibition of *N. mucosa* L-lactate utilization and pyrimidine biosynthesis in synthetic sputum

Supplemental material mbio.01948-25-s0004.docx Supplemental figure legends and Table S1.

ASM does not own the copyrights to Supplemental Material that may be linked to, or accessed through, an article. The authors have granted ASM a non-exclusive, world-wide license to publish the Supplemental Material files. Please contact the corresponding author directly for reuse.

## References

[1] Escapa IF, Chen T, Huang Y, Gajare P, Dewhirst FE, Lemon KP. 2018. New insights into human nostril microbiome from the expanded human oral microbiome database (ehomd): a resource for the microbiome of the human aerodigestive tract. mSystems 3:e00187-18. doi:10.1128/mSystems.00187-18 PMC6280432 30534599

[2] Hajishengallis G, Chavakis T. 2021. Local and systemic mechanisms linking periodontal disease and inflammatory comorbidities. Nat Rev Immunol 21:426–440. doi:10.1038/s41577-020-00488-6 33510490 PMC7841384

[3] Pussinen PJ, Kopra E, Pietiäinen M, Lehto M, Zaric S, Paju S, Salminen A. 2022. Periodontitis and cardiometabolic disorders: the role of lipopolysaccharide and endotoxemia. Periodontol 2000 89:19–40. doi:10.1111/prd.12433 35244966 PMC9314839

[4] Rosier BT, Johnston W, Carda-Diéguez M, Simpson A, Cabello-Yeves E, Piela K, Reilly R, Artacho A, Easton C, Burleigh M, Culshaw S, Mira A. 2024. Nitrate reduction capacity of the oral microbiota is impaired in periodontitis: potential implications for systemic nitric oxide availability. Int J Oral Sci 16:1. doi:10.1038/s41368-023-00266-9 38177101 PMC10767001

[5] Xiao L, Huang L, Zhou X, Zhao D, Wang Y, Min H, Song S, Sun W, Gao Q, Hu Q, Xie S. 2021. Experimental periodontitis deteriorated atherosclerosis associated with trimethylamine n-oxide metabolism in mice. Front Cell Infect Microbiol 11:820535. doi:10.3389/fcimb.2021.820535 35118014 PMC8804528

[6] Li X, Wang H, Yu X, Saha G, Kalafati L, Ioannidis C, Mitroulis I, Netea MG, Chavakis T, Hajishengallis G. 2022. Maladaptive innate immune training of myelopoiesis links inflammatory comorbidities. Cell 185:1709–1727. doi:10.1016/j.cell.2022.03.043 35483374 PMC9106933

[7] Atarashi K, Suda W, Luo C, Kawaguchi T, Motoo I, Narushima S, Kiguchi Y, Yasuma K, Watanabe E, Tanoue T, et al.. 2017. Ectopic colonization of oral bacteria in the intestine drives T_H_1 cell induction and inflammation. Science 358:359–365. doi:10.1126/science.aan4526 29051379 PMC5682622

[8] Kitamoto S, Nagao-Kitamoto H, Jiao Y, Gillilland MG III, Hayashi A, Imai J, Sugihara K, Miyoshi M, Brazil JC, Kuffa P, Hill BD, Rizvi SM, Wen F, Bishu S, Inohara N, Eaton KA, Nusrat A, Lei YL, Giannobile WV, Kamada N. 2020. The intermucosal connection between the mouth and gut in commensal pathobiont-driven colitis. Cell 182:447–462. doi:10.1016/j.cell.2020.05.048 32758418 PMC7414097

[9] Rahamat-Langendoen JC, van Vonderen MGA, Engström LJ, Manson WL, van Winkelhoff AJ, Mooi-Kokenberg EANM. 2011. Brain abscess associated with Aggregatibacter actinomycetemcomitans: case report and review of literature. J Clin Periodontol 38:702–706. doi:10.1111/j.1600-051X.2011.01737.x 21539594

[10] Webb BJ, Fisher MA, Tinker N. 2025. Re: “HANCEK” infective endocarditis: a case for including Neisseria elongata in the HACEK group. Clin Microbiol Infect 31:651–653. doi:10.1016/j.cmi.2024.10.015 39447747

[11] Walsh L, Clark SA, Derrick JP, Borrow R. 2023. Beyond the usual suspects: reviewing infections caused by typically-commensal Neisseria species. J Infect 87:479–489. doi:10.1016/j.jinf.2023.09.007 37797844

[12] Li L, Mac Aogáin M, Xu T, Jaggi TK, Chan LLY, Qu J, Wei L, Liao S, Cheng HS, Keir HR, et al.. 2022. Neisseria species as pathobionts in bronchiectasis. Cell Host Microbe 30:1311–1327. doi:10.1016/j.chom.2022.08.005 36108613

[13] Gris P, Vincke G, Delmez JP, Dierckx JP. 1989. Neisseria sicca pneumonia and bronchiectasis. Eur Respir J 2:685–687. doi:10.1183/09031936.93.02070685 2776875

[14] Giacomini JJ, Torres-Morales J, Dewhirst FE, Borisy GG, Mark Welch JL. 2025. Spatial ecology of the Neisseriaceae family in the human oral cavity. Microbiol Spectr 13:e0327524. doi:10.1128/spectrum.03275-24 40197060 PMC12054151

[15] Mark Welch JL, Dewhirst FE, Borisy GG. 2019. Biogeography of the oral microbiome: the site-specialist hypothesis. Annu Rev Microbiol 73:335–358. doi:10.1146/annurev-micro-090817-062503 31180804 PMC7153577

[16] Scannapieco FA, Giuliano KK, Baker D. 2022. Oral health status and the etiology and prevention of nonventilator hospital‐associated pneumonia. Periodontol 2000 89:51–58. doi:10.1111/prd.12423 35244952

[17] Mammen MJ, Scannapieco FA, Sethi S. 2020. Oral‐lung microbiome interactions in lung diseases. Periodontol 2000 83:234–241. doi:10.1111/prd.12301 32385873

[18] Dong J, Li W, Wang Q, Chen J, Zu Y, Zhou X, Guo Q. 2021. Relationships between oral microecosystem and respiratory diseases. Front Mol Biosci 8:718222. doi:10.3389/fmolb.2021.718222 35071321 PMC8767498

[19] Molina A, Huck O, Herrera D, Montero E. 2023. The association between respiratory diseases and periodontitis: a systematic review and meta-analysis. J Clin Periodontol 50:842–887. doi:10.1111/jcpe.13767 36606394

[20] He J, Mao N, Lyu W, Zhou S, Zhang Y, Liu Z, Xu Z. 2024. Association between oral microbiome and five types of respiratory infections: a two-sample Mendelian randomization study in east Asian population. Front Microbiol 15:1392473. doi:10.3389/fmicb.2024.1392473 38659993 PMC11039966

[21] Tiew PY, Jaggi TK, Chan LLY, Chotirmall SH. 2021. The airway microbiome in COPD, bronchiectasis and bronchiectasis-COPD overlap. Clin Respir J 15:123–133. doi:10.1111/crj.13294 33063421

[22] van der Gast CJ, Walker AW, Stressmann FA, Rogers GB, Scott P, Daniels TW, Carroll MP, Parkhill J, Bruce KD. 2011. Partitioning core and satellite taxa from within cystic fibrosis lung bacterial communities. ISME J 5:780–791. doi:10.1038/ismej.2010.175 21151003 PMC3105771

[23] Rogers GB, van der Gast CJ, Cuthbertson L, Thomson SK, Bruce KD, Martin ML, Serisier DJ. 2013. Clinical measures of disease in adult non-CF bronchiectasis correlate with airway microbiota composition. Thorax 68:731–737. doi:10.1136/thoraxjnl-2012-203105 23564400

[24] O’Toole GA. 2018. Cystic fibrosis airway microbiome: overturning the old, opening the way for the new. J Bacteriol 200:e00561-17. doi:10.1128/JB.00561-17 29084859 PMC5786703

[25] Filkins LM, Hampton TH, Gifford AH, Gross MJ, Hogan DA, Sogin ML, Morrison HG, Paster BJ, O’Toole GA. 2012. Prevalence of streptococci and increased polymicrobial diversity associated with cystic fibrosis patient stability. J Bacteriol 194:4709–4717. doi:10.1128/JB.00566-12 22753064 PMC3415522

[26] Baty JJ, Stoner SN, McDaniel MS, Huffines JT, Edmonds SE, Evans NJ, Novak L, Scoffield JA. 2023. An oral commensal attenuates Pseudomonas aeruginosa-induced airway inflammation and modulates nitrite flux in respiratory epithelium. Microbiol Spectr 11:e0219823. doi:10.1128/spectrum.02198-23 37800950 PMC10715204

[27] Segal LN, Clemente JC, Tsay J-CJ, Koralov SB, Keller BC, Wu BG, Li Y, Shen N, Ghedin E, Morris A, Diaz P, Huang L, Wikoff WR, Ubeda C, Artacho A, Rom WN, Sterman DH, Collman RG, Blaser MJ, Weiden MD. 2016. Enrichment of the lung microbiome with oral taxa is associated with lung inflammation of a Th17 phenotype. Nat Microbiol 1:16031. doi:10.1038/nmicrobiol.2016.31 27572644 PMC5010013

[28] Konovalovas A, Armalytė J, Klimkaitė L, Liveikis T, Jonaitytė B, Danila E, Bironaitė D, Mieliauskaitė D, Bagdonas E, Aldonytė R. 2024. Insights into respiratory microbiome composition and systemic inflammatory biomarkers of bronchiectasis patients. Microbiol Spectr 12:e0414423. doi:10.1128/spectrum.04144-23 39535197 PMC11619244

[29] Criss AK, Seifert HS. 2012. A bacterial siren song: intimate interactions between Neisseria and neutrophils. Nat Rev Microbiol 10:178–190. doi:10.1038/nrmicro2713 22290508 PMC3569855

[30] Liu G, Tang CM, Exley RM. 2015. Non-pathogenic Neisseria: members of an abundant, multi-habitat, diverse genus. Microbiology (Reading) 161:1297–1312. doi:10.1099/mic.0.000086 25814039

[31] Weyand NJ. 2017. Neisseria models of infection and persistence in the upper respiratory tract. Pathog Dis 75. doi:10.1093/femspd/ftx031 28369241

[32] Custodio R, Johnson E, Liu G, Tang CM, Exley RM. 2020. Commensal Neisseria cinerea impairs Neisseria meningitidis microcolony development and reduces pathogen colonisation of epithelial cells. PLoS Pathog 16:e1008372. doi:10.1371/journal.ppat.1008372 32208456 PMC7092958

[33] Deasy AM, Guccione E, Dale AP, Andrews N, Evans CM, Bennett JS, Bratcher HB, Maiden MCJ, Gorringe AR, Read RC. 2015. Nasal inoculation of the commensal Neisseria lactamica inhibits carriage of neisseria meningitidis by young adults: a controlled human infection study. Clin Infect Dis 60:1512–1520. doi:10.1093/cid/civ098 25814628

[34] Alles M, Gunasena M, Zia T, D’Mello A, Bhattarai S, Mulhern W, Terry L, Scherger T, Wijeratne S, Singh S, Wijeratne AJ, Kasturiratna D, Tettelin H, Weyand NJ, Liyanage NPM. 2024. Unveiling the immune dynamics of Neisseria persistent oral colonization. Infect Immun 92:e0004824. doi:10.1128/iai.00048-24 38814083 PMC11238562

[35] Zhu W, Cardenas-Alvarez MX, Tomberg J, Little MB, Duncan JA, Nicholas RA. 2023. Commensal Neisseria species share immune suppressive mechanisms with Neisseria gonorrhoeae. PLoS One 18:e0284062. doi:10.1371/journal.pone.0284062 37027389 PMC10081783

[36] Donati C, Zolfo M, Albanese D, Tin Truong D, Asnicar F, Iebba V, Cavalieri D, Jousson O, De Filippo C, Huttenhower C, Segata N. 2016. Uncovering oral Neisseria tropism and persistence using metagenomic sequencing. Nat Microbiol 1:16070. doi:10.1038/nmicrobiol.2016.70 27572971

[37] Xue Q, Xie Y, He Y, Yu Y, Fang G, Yu W, Wu J, Li J, Zhao L, Deng X, Li R, Wang F, Zheng Y, Gao Z. 2023. Lung microbiome and cytokine profiles in different disease states of COPD: a cohort study. Sci Rep 13:5715. doi:10.1038/s41598-023-32901-0 37029178 PMC10080507

[38] Rhodes KA, Ma MC, Rendón MA, So M. 2022. Neisseria genes required for persistence identified via in vivo screening of a transposon mutant library. PLoS Pathog 18:e1010497. doi:10.1371/journal.ppat.1010497 35580146 PMC9140248

[39] Smith H, Tang CM, Exley RM. 2007. Effect of host lactate on gonococci and meningococci: new concepts on the role of metabolites in pathogenicity. Infect Immun 75:4190–4198. doi:10.1128/IAI.00117-07 17562766 PMC1951187

[40] Potter AD, Criss AK. 2024. Dinner date: Neisseria gonorrhoeae central carbon metabolism and pathogenesis. Emerg Top Life Sci 8:15–28. doi:10.1042/ETLS20220111 37144661 PMC10625648

[41] Hoshino E, Yamada T, Araya S. 1976. Lactate degradation by a strain of Neisseria isolated from human dental plaque. Arch Oral Biol 21:677–683. doi:10.1016/0003-9969(76)90142-4 1069579

[42] Wright TK, Gibson PG, Simpson JL, McDonald VM, Wood LG, Baines KJ. 2016. Neutrophil extracellular traps are associated with inflammation in chronic airway disease. Respirology 21:467–475. doi:10.1111/resp.12730 26804470

[43] Dwyer M, Shan Q, D’Ortona S, Maurer R, Mitchell R, Olesen H, Thiel S, Huebner J, Gadjeva M. 2014. Cystic fibrosis sputum DNA has NETosis characteristics and neutrophil extracellular trap release is regulated by macrophage migration-inhibitory factor. J Innate Immun 6:765–779. doi:10.1159/000363242 24862346 PMC4201867

[44] Fredman G, Kolpen M, Hertz FB, Petersen PT, Jensen AV, Baunbaek-Egelund G, Ravn P, Jensen PØ, Faurholt-Jepsen D. 2019. The inflamed sputum in lower respiratory tract infection: l-lactate levels are correlated to neutrophil accumulation. APMIS 127:72–79. doi:10.1111/apm.12913 30614067 PMC7159756

[45] Britigan BE, Klapper D, Svendsen T, Cohen MS. 1988. Phagocyte-derived lactate stimulates oxygen consumption by Neisseria gonorrhoeae. an unrecognized aspect of the oxygen metabolism of phagocytosis. J Clin Invest 81:318–324. doi:10.1172/JCI113323 3123517 PMC329573

[46] Zhou Y, He X, Tang J, Zhang D, Liu Y, Xue Y, Jiang N, Zhang J, Wang X. 2024. Total sputum nitrate/nitrite is associated with exacerbations and Pseudomonas aeruginosa colonisation in bronchiectasis. ERJ Open Res 10:01045-2023. doi:10.1183/23120541.01045-2023 39040581 PMC11261385

[47] Linnane SJ, Keatings VM, Costello CM, Moynihan JB, O’Connor CM, Fitzgerald MX, McLoughlin P. 1998. Total sputum nitrate plus nitrite is raised during acute pulmonary infection in cystic fibrosis. Am J Respir Crit Care Med 158:207–212. doi:10.1164/ajrccm.158.1.9707096 9655731

[48] Mac Aogáin M, Narayana JK, Tiew PY, Ali NABM, Yong VFL, Jaggi TK, Lim AYH, Keir HR, Dicker AJ, Thng KX, et al.. 2021. Integrative microbiomics in bronchiectasis exacerbations. Nat Med 27:688–699. doi:10.1038/s41591-021-01289-7 33820995

[49] Widder S, Zhao J, Carmody LA, Zhang Q, Kalikin LM, Schloss PD, LiPuma JJ. 2022. Association of bacterial community types, functional microbial processes and lung disease in cystic fibrosis airways. ISME J 16:905–914. doi:10.1038/s41396-021-01129-z 34689185 PMC8941020

[50] Mac Aogáin M, Tiew PY, Jaggi TK, Narayana JK, Singh S, Hansbro PM, Segal LN, Chotirmall SH. 2024. Targeting respiratory microbiomes in COPD and bronchiectasis. Expert Rev Respir Med 18:111–125. doi:10.1080/17476348.2024.2355155 38743428

[51] Turner KH, Wessel AK, Palmer GC, Murray JL, Whiteley M. 2015. Essential genome of Pseudomonas aeruginosa in cystic fibrosis sputum. Proc Natl Acad Sci USA 112:4110–4115. doi:10.1073/pnas.1419677112 25775563 PMC4386324

[52] Palmer KL, Aye LM, Whiteley M. 2007. Nutritional cues control Pseudomonas aeruginosa multicellular behavior in cystic fibrosis sputum. J Bacteriol 189:8079–8087. doi:10.1128/JB.01138-07 17873029 PMC2168676

[53] Cornforth DM, Diggle FL, Melvin JA, Bomberger JM, Whiteley M. 2020. Quantitative framework for model evaluation in microbiology research using Pseudomonas aeruginosa and cystic fibrosis infection as a test case. mBio 11:e03042-19. doi:10.1128/mBio.03042-19 31937646 PMC6960289

[54] Lewin GR, Kapur A, Cornforth DM, Duncan RP, Diggle FL, Moustafa DA, Harrison SA, Skaar EP, Chazin WJ, Goldberg JB, Bomberger JM, Whiteley M. 2023. Application of a quantitative framework to improve the accuracy of a bacterial infection model. Proc Natl Acad Sci USA 120:e2221542120. doi:10.1073/pnas.2221542120 37126703 PMC10175807

[55] Duncan RP, Moustafa DA, Lewin GR, Diggle FL, Bomberger JM, Whiteley M, Goldberg JB. 2024. Improvement of a mouse infection model to capture Pseudomonas aeruginosa chronic physiology in cystic fibrosis. Proc Natl Acad Sci USA 121:e2406234121. doi:10.1073/pnas.2406234121 39102545 PMC11331117

[56] Cain AK, Barquist L, Goodman AL, Paulsen IT, Parkhill J, van Opijnen T. 2020. A decade of advances in transposon-insertion sequencing. Nat Rev Genet 21:526–540. doi:10.1038/s41576-020-0244-x 32533119 PMC7291929

[57] Winter SE, Winter MG, Xavier MN, Thiennimitr P, Poon V, Keestra AM, Laughlin RC, Gomez G, Wu J, Lawhon SD, Popova IE, Parikh SJ, Adams LG, Tsolis RM, Stewart VJ, Bäumler AJ. 2013. Host-derived nitrate boosts growth of E. coli in the inflamed gut. Science 339:708–711. doi:10.1126/science.1232467 23393266 PMC4004111

[58] Palmer KL, Brown SA, Whiteley M. 2007. Membrane-bound nitrate reductase is required for anaerobic growth in cystic fibrosis sputum. J Bacteriol 189:4449–4455. doi:10.1128/JB.00162-07 17400735 PMC1913347

[59] Nakatsuka Y, Matsumoto M, Inohara N, Núñez G. 2023. Pseudomonas aeruginosa hijacks the murine nitric oxide metabolic pathway to evade killing by neutrophils in the lung. Cell Rep 42:112973. doi:10.1016/j.celrep.2023.112973 37561628

[60] Zhu W, Winter MG, Byndloss MX, Spiga L, Duerkop BA, Hughes ER, Büttner L, de Lima Romão E, Behrendt CL, Lopez CA, Sifuentes-Dominguez L, Huff-Hardy K, Wilson RP, Gillis CC, Tükel Ç, Koh AY, Burstein E, Hooper LV, Bäumler AJ, Winter SE. 2018. Precision editing of the gut microbiota ameliorates colitis. Nature 553:208–211. doi:10.1038/nature25172 29323293 PMC5804340

[61] Altenburg J, de Graaff CS, Stienstra Y, Sloos JH, van Haren EHJ, Koppers RJH, van der Werf TS, Boersma WG. 2013. Effect of azithromycin maintenance treatment on infectious exacerbations among patients with non-cystic fibrosis bronchiectasis: the BAT randomized controlled trial. JAMA 309:1251–1259. doi:10.1001/jama.2013.1937 23532241

[62] Chalmers JD, Boersma W, Lonergan M, Jayaram L, Crichton ML, Karalus N, Taylor SL, Martin ML, Burr LD, Wong C, Altenburg J. 2019. Long-term macrolide antibiotics for the treatment of bronchiectasis in adults: an individual participant data meta-analysis. Lancet Respir Med 7:845–854. doi:10.1016/S2213-2600(19)30191-2 31405828

[63] Day MJ, Jacobsson S, Spiteri G, Kulishev C, Sajedi N, Woodford N, Blumel B, Werf MJ, Amato-Gauci AJ, Unemo M, Cole MJ, Gn E. 2019. Significant increase in azithromycin “resistance” and susceptibility to ceftriaxone and cefixime in Neisseria gonorrhoeae isolates in 26 European countries, 2019. BMC Infect Dis 22:524. doi:10.1186/s12879-022-07509-w PMC9171984 35672671

[64] Vanbaelen T, Van Dijck C, Laumen J, Gonzalez N, De Baetselier I, Manoharan-Basil SS, De Block T, Kenyon C. 2022. Global epidemiology of antimicrobial resistance in commensal Neisseria species: a systematic review. Int J Med Microbiol 312:151551. doi:10.1016/j.ijmm.2022.151551 35231823

[65] Hughes D, Andersson DI. 2017. Environmental and genetic modulation of the phenotypic expression of antibiotic resistance. FEMS Microbiol Rev 41:374–391. doi:10.1093/femsre/fux004 28333270 PMC5435765

[66] Ghuneim L-AJ, Raghuvanshi R, Neugebauer KA, Guzior DV, Christian MH, Schena B, Feiner JM, Castillo-Bahena A, Mielke J, McClelland M, Conrad D, Klapper I, Zhang T, Quinn RA. 2022. Complex and unexpected outcomes of antibiotic therapy against a polymicrobial infection. ISME J 16:2065–2075. doi:10.1038/s41396-022-01252-5 35597889 PMC9381758

[67] VERON M, THIBAULT P, SECOND L. 1959. Neisseria mucosa (Diplococcus mucosus Lingelsheim). I. Bacteriological description and study of its pathogenicity. Ann Inst Pasteur (Paris) 97:497–510. 13841905

[68] Kersh EN, Allen V, Ransom E, Schmerer M, Cyr S, Workowski K, Weinstock H, Patel J, Ferraro MJ. 2020. Rationale for a Neisseria gonorrhoeae susceptible-only interpretive breakpoint for azithromycin. Clin Infect Dis 70:798–804. doi:10.1093/cid/ciz292 30963175 PMC6785360

[69] Haworth CS, Floto RA. 2022. Antibiotic management in bronchiectasis. Clin Chest Med 43:165–177. doi:10.1016/j.ccm.2021.11.009 35236556

[70] Cowley ES, Kopf SH, LaRiviere A, Ziebis W, Newman DK. 2015. Pediatric cystic fibrosis sputum can be chemically dynamic, anoxic, and extremely reduced due to hydrogen sulfide formation. mBio 6:e00767. doi:10.1128/mBio.00767-15 26220964 PMC4551978

[71] Tunney MM, Field TR, Moriarty TF, Patrick S, Doering G, Muhlebach MS, Wolfgang MC, Boucher R, Gilpin DF, McDowell A, Elborn JS. 2008. Detection of anaerobic bacteria in high numbers in sputum from patients with cystic fibrosis. Am J Respir Crit Care Med 177:995–1001. doi:10.1164/rccm.200708-1151OC 18263800

[72] Rock JD, Mahnane MR, Anjum MF, Shaw JG, Read RC, Moir JWB. 2005. The pathogen Neisseria meningitidis requires oxygen, but supplements growth by denitrification. Nitrite, nitric oxide and oxygen control respiratory flux at genetic and metabolic levels. Mol Microbiol 58:800–809. doi:10.1111/j.1365-2958.2005.04866.x 16238628

[73] Dillard JP, Chan JM. 2024. Genetic manipulation of Neisseria gonorrhoeae and commensal neisseria species. Curr Protoc 4:e70000. doi:10.1002/cpz1.70000 39228292 PMC11658436

[74] Kirby JR. 2007. In vivo mutagenesis using EZ-Tn5. Methods Enzymol 421:17–21. doi:10.1016/S0076-6879(06)21003-6 17352911

[75] Stubenrauch CJ, Lithgow T. 2019. The TAM: a translocation and assembly module of the β-barrel assembly machinery in bacterial outer membranes. EcoSal Plus 8. doi:10.1128/ecosalplus.ESP-0036-2018 PMC11573294 30816086

[76] Schaub RE, Dillard JP. 2019. The pathogenic Neisseria use a streamlined set of peptidoglycan degradation proteins for peptidoglycan remodeling, recycling, and toxic fragment release. Front Microbiol 10:73. doi:10.3389/fmicb.2019.00073 30766523 PMC6365954

[77] Zarantonelli L, Borthagaray G, Lee EH, Shafer WM. 1999. Decreased azithromycin susceptibility of Neisseria gonorrhoeae due to mtrR mutations. Antimicrob Agents Chemother 43:2468–2472. doi:10.1128/AAC.43.10.2468 10508026 PMC89502

[78] Wadsworth CB, Arnold BJ, Sater MRA, Grad YH. 2018. Azithromycin resistance through interspecific acquisition of an epistasis-dependent efflux pump component and transcriptional regulator in Neisseria gonorrhoeae. mBio 9:e01419-18. doi:10.1128/mBio.01419-18 30087172 PMC6083905

[79] Galperin MY, Kristensen DM, Makarova KS, Wolf YI, Koonin EV. 2019. Microbial genome analysis: the COG approach. Brief Bioinform 20:1063–1070. doi:10.1093/bib/bbx117 28968633 PMC6781585

[80] Peekhaus N, Conway T. 1998. What’s for dinner?: entner-doudoroff metabolism in Escherichia coli. J Bacteriol 180:3495–3502. doi:10.1128/JB.180.14.3495-3502.1998 9657988 PMC107313

[81] Vasiljevs S, Gupta A, Baines D. 2023. Effect of glucose on growth and co-culture of Staphylococcus aureus and Pseudomonas aeruginosa in artificial sputum medium. Heliyon 9:e21469. doi:10.1016/j.heliyon.2023.e21469 37908712 PMC10613906

[82] Pajon C, Fortoul MC, Diaz-Tang G, Marin Meneses E, Kalifa AR, Sevy E, Mariah T, Toscan B, Marcelin M, Hernandez DM, Marzouk MM, Lopatkin AJ, Eldakar OT, Smith RP. 2023. Interactions between metabolism and growth can determine the co-existence of Staphylococcus aureus and Pseudomonas aeruginosa Elife 12:e83664. doi:10.7554/eLife.83664 37078696 PMC10174691

[83] Exley RM, Wu H, Shaw J, Schneider MC, Smith H, Jerse AE, Tang CM. 2007. Lactate acquisition promotes successful colonization of the murine genital tract by Neisseria gonorrhoeae. Infect Immun 75:1318–1324. doi:10.1128/IAI.01530-06 17158905 PMC1828543

[84] Exley RM, Goodwin L, Mowe E, Shaw J, Smith H, Read RC, Tang CM. 2005. Neisseria meningitidis lactate permease is required for nasopharyngeal colonization. Infect Immun 73:5762–5766. doi:10.1128/IAI.73.9.5762-5766.2005 16113293 PMC1231078

[85] Chen NH, Ong C-LY, O’sullivan J, Ibranovic I, Davey K, Edwards JL, McEwan AG. 2020. Two distinct l-lactate dehydrogenases play a role in the survival of Neisseria gonorrhoeae in cervical epithelial cells. J Infect Dis 221:449–453. doi:10.1093/infdis/jiz468 31541571 PMC7530546

[86] Kaiser JC, Heinrichs DE. 2018. Branching out: alterations in bacterial physiology and virulence due to branched-chain amino acid deprivation. mBio 9:e01188-18. doi:10.1128/mBio.01188-18 30181248 PMC6123439

[87] Goncheva MI, Chin D, Heinrichs DE. 2022. Nucleotide biosynthesis: the base of bacterial pathogenesis. Trends Microbiol 30:793–804. doi:10.1016/j.tim.2021.12.007 35074276

[88] Barth KR, Isabella VM, Clark VL. 2009. Biochemical and genomic analysis of the denitrification pathway within the genus Neisseria. Microbiology (Reading) 155:4093–4103. doi:10.1099/mic.0.032961-0 19762442 PMC2788039

[89] Seth D, Hausladen A, Stamler JS. 2020. Anaerobic transcription by OxyR: a novel paradigm for nitrosative stress. Antioxid Redox Signal 32:803–816. doi:10.1089/ars.2019.7921 31691575 PMC7074925

[90] Rosier BT, Takahashi N, Zaura E, Krom BP, MartÍnez-Espinosa RM, van Breda SGJ, Marsh PD, Mira A. 2022. The importance of nitrate reduction for oral health. J Dent Res 101:887–897. doi:10.1177/00220345221080982 35196931

[91] Brown SA, Whiteley M. 2009. Characterization of the L-lactate dehydrogenase from Aggregatibacter actinomycetemcomitans. PLoS One 4:e7864. doi:10.1371/journal.pone.0007864 19924225 PMC2773005

[92] Jatana S, Homer CR, Madajka M, Ponti AK, Kabi A, Papay F, McDonald C. 2018. Pyrimidine synthesis inhibition enhances cutaneous defenses against antibiotic resistant bacteria through activation of NOD2 signaling. Sci Rep 8:8708. doi:10.1038/s41598-018-27012-0 29880914 PMC5992176

[93] Taylor SJ, Winter MG, Gillis CC, Silva LA da, Dobbins AL, Muramatsu MK, Jimenez AG, Chanin RB, Spiga L, Llano EM, Rojas VK, Kim J, Santos RL, Zhu W, Winter SE. 2022. Colonocyte-derived lactate promotes E. coli fitness in the context of inflammation-associated gut microbiota dysbiosis. Microbiome 10:200. doi:10.1186/s40168-022-01389-7 36434690 PMC9701030

[94] Jakubovics NS. 2015. Saliva as the sole nutritional source in the development of multispecies communities in dental plaque. Microbiol Spectr 3. doi:10.1128/microbiolspec.MBP-0013-2014 26185065

[95] Rosier BT, Buetas E, Moya-Gonzalvez EM, Artacho A, Mira A. 2020. Nitrate as a potential prebiotic for the oral microbiome. Sci Rep 10:12895. doi:10.1038/s41598-020-69931-x 32732931 PMC7393384

[96] Mazurel D, Carda-Diéguez M, Langenburg T, Žiemytė M, Johnston W, Martínez CP, Albalat F, Llena C, Al-Hebshi N, Culshaw S, Mira A, Rosier BT. 2023. Nitrate and a nitrate-reducing Rothia aeria strain as potential prebiotic or synbiotic treatments for periodontitis. NPJ Biofilms Microbiomes 9:40. doi:10.1038/s41522-023-00406-3 37330520 PMC10276839

[97] Rojas-Tapias DF, Brown EM, Temple ER, Onyekaba MA, Mohamed AMT, Duncan K, Schirmer M, Walker RL, Mayassi T, Pierce KA, Ávila-Pacheco J, Clish CB, Vlamakis H, Xavier RJ. 2022. Inflammation-associated nitrate facilitates ectopic colonization of oral bacterium Veillonella parvula in the intestine. Nat Microbiol 7:1673–1685. doi:10.1038/s41564-022-01224-7 36138166 PMC9728153

[98] Sohn J, Li L, Zhang L, Genco RJ, Falkner KL, Tettelin H, Rowsam AM, Smiraglia DJ, Novak JM, Diaz PI, Sun Y, Kirkwood KL. 2023. Periodontal disease is associated with increased gut colonization of pathogenic Haemophilus parainfluenzae in patients with Crohn’s disease. Cell Rep 42:112120. doi:10.1016/j.celrep.2023.112120 36774550 PMC10415533

[99] Gao B, Gallagher T, Zhang Y, Elbadawi-Sidhu M, Lai Z, Fiehn O, Whiteson KL. 2018. Tracking polymicrobial metabolism in cystic fibrosis airways: Pseudomonas aeruginosa metabolism and physiology are influenced by rothia mucilaginosa-derived metabolites. mSphere 3. doi:10.1128/mSphere.00151-18 PMC5917424 29695623

[100] Chavez-Arroyo A, Radlinski LC, Bäumler AJ. 2025. Principles of gut microbiota assembly. Trends Microbiol 33:718–726. doi:10.1016/j.tim.2025.02.014 40089422

[101] Morales LD, Av-Gay Y, Murphy MEP. 2023. Acidic pH modulates Burkholderia cenocepacia antimicrobial susceptibility in the cystic fibrosis nutritional environment. Microbiol Spectr 11:e0273123. doi:10.1128/spectrum.02731-23 37966209 PMC10714822

[102] Baker EJ, Allcott G, Molloy A, Cox JAG. 2024. Cystic fibrosis sputum media induces an overall loss of antibiotic susceptibility in Mycobacterium abscessus. NPJ Antimicrob Resist 2:34. doi:10.1038/s44259-024-00054-3 39843503 PMC11721417

[103] Zhu W, Miyata N, Winter MG, Arenales A, Hughes ER, Spiga L, Kim J, Sifuentes-Dominguez L, Starokadomskyy P, Gopal P, Byndloss MX, Santos RL, Burstein E, Winter SE. 2019. Editing of the gut microbiota reduces carcinogenesis in mouse models of colitis-associated colorectal cancer. J Exp Med 216:2378–2393. doi:10.1084/jem.20181939 31358565 PMC6781011

[104] Bolt AM, Mann KK. 2016. Tungsten: an emerging toxicant, alone or in combination. Curr Environ Health Rep 3:405–415. doi:10.1007/s40572-016-0106-z 27678292

[105] Socransky SS, Dzink JL, Smith CM. 1985. Chemically defined medium for oral microorganisms. J Clin Microbiol 22:303–305. doi:10.1128/jcm.22.2.303-305.1985 3897273 PMC268381

[106] Brown SA, Whiteley M. 2007. A novel exclusion mechanism for carbon resource partitioning in Aggregatibacter actinomycetemcomitans. J Bacteriol 189:6407–6414. doi:10.1128/JB.00554-07 17586632 PMC1951915

[107] Mahen KK, Markley L, Bogart J, Klatka H, Krishna V, Maytin EV, Stark GR, McDonald C. 2023. Topical N-phosphonacetyl-l-aspartate is a dual action candidate for treating non-melanoma skin cancer. Exp Dermatol 32:1485–1497. doi:10.1111/exd.14853 37309615 PMC10527533

[108] Hung CS, Dodson KW, Hultgren SJ. 2009. A murine model of urinary tract infection. Nat Protoc 4:1230–1243. doi:10.1038/nprot.2009.116 19644462 PMC2963178

[109] Gallagher LA, Bailey J, Manoil C. 2020. Ranking essential bacterial processes by speed of mutant death. Proc Natl Acad Sci USA 117:18010–18017. doi:10.1073/pnas.2001507117 32665440 PMC7395459

[110] Brogan JM, Lally ET, Demuth DR. 1996. Construction of pYGK, an Actinobacillus actinomycetemcomitans-Escherichia coli shuttle vector. Gene 169:141–142. doi:10.1016/0378-1119(95)00792-x 8635742

[111] Lewin GR, Stacy A, Michie KL, Lamont RJ, Whiteley M. 2019. Large-scale identification of pathogen essential genes during coinfection with sympatric and allopatric microbes. Proc Natl Acad Sci USA 116:19685–19694. doi:10.1073/pnas.1907619116 31427504 PMC6765283

[112] Stacy A, Fleming D, Lamont RJ, Rumbaugh KP, Whiteley M. 2016. A commensal bacterium promotes virulence of an opportunistic pathogen via cross-respiration. mBio 7:e00782-16. doi:10.1128/mBio.00782-16 27353758 PMC4916382

[113] Langmead B, Salzberg SL. 2012. Fast gapped-read alignment with Bowtie 2. Nat Methods 9:357–359. doi:10.1038/nmeth.1923 22388286 PMC3322381

[114] Liao Y, Smyth GK, Shi W. 2019. The R package Rsubread is easier, faster, cheaper and better for alignment and quantification of RNA sequencing reads. Nucleic Acids Res 47:e47. doi:10.1093/nar/gkz114 30783653 PMC6486549

[115] Love MI, Huber W, Anders S. 2014. Moderated estimation of fold change and dispersion for RNA-seq data with DESeq2. Genome Biol 15:550. doi:10.1186/s13059-014-0550-8 25516281 PMC4302049

[116] Quinlan AR, Hall IM. 2010. BEDTools: a flexible suite of utilities for comparing genomic features. Bioinformatics 26:841–842. doi:10.1093/bioinformatics/btq033 20110278 PMC2832824

[117] Moriya Y, Itoh M, Okuda S, Yoshizawa AC, Kanehisa M. 2007. KAAS: an automatic genome annotation and pathway reconstruction server. Nucleic Acids Res 35:W182–W185. doi:10.1093/nar/gkm321 17526522 PMC1933193

[118] Narayanan AM, Ramsey MM, Stacy A, Whiteley M. 2017. Defining genetic fitness determinants and creating genomic resources for an oral pathogen. Appl Environ Microbiol 83:e00797-17. doi:10.1128/AEM.00797-17 28476775 PMC5494627

[119] Qvarnstrom Y, Swedberg G. 2006. Variations in gene organization and DNA uptake signal sequence in the folP region between commensal and pathogenic Neisseria species. BMC Microbiol 6:11. doi:10.1186/1471-2180-6-11 16503987 PMC1431543

