# Supplementary figures and images for: Metabolic strategies that enable oral commensal persistence in a lower airway environment
Source: mBio. 2025 Sep 22;16(11):e01948-25. doi: 10.1128/mbio.01948-25 (PMC12607641; doi:10.1128/mbio.01948-25)

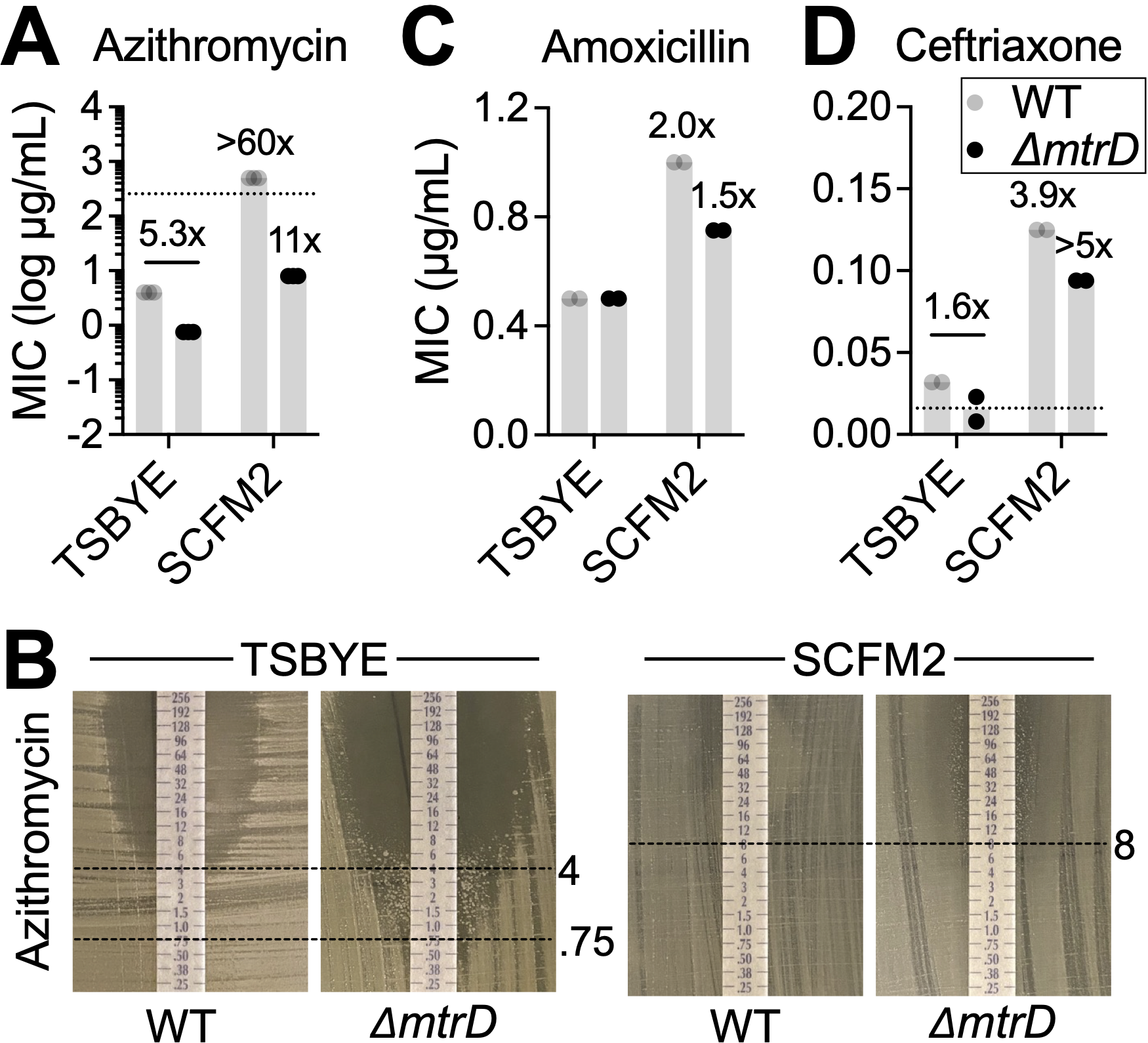

Supplement: Fig. S1 — The Mtr efflux pump partially mediates the enhancement of N. mucosa antibiotic resistance on SCFM2. [file mbio.01948-25-s0002.tiff]

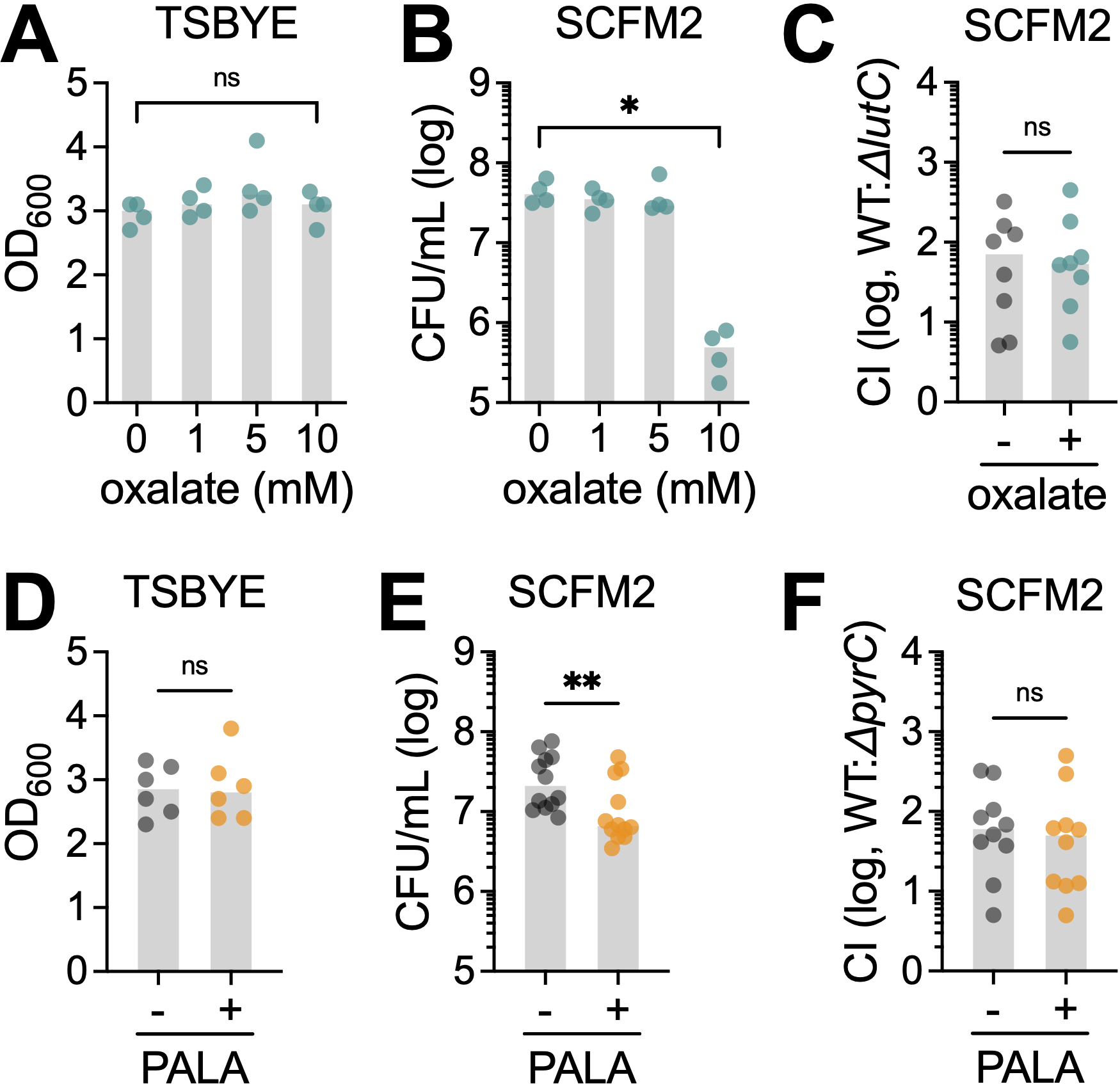

Supplement: Fig. S2 — Inhibition of N. mucosa L-lactate utilization and pyrimidine biosynthesis in synthetic sputum [file mbio.01948-25-s0003.tiff]
