# Supplementary material for: Metabolic strategies that enable oral commensal persistence in a lower airway environment
Source: mBio. 2025 Sep 22;16(11):e01948-25. doi: 10.1128/mbio.01948-25 (PMC12607641; doi:10.1128/mbio.01948-25)
Supplement: Supplemental material — Supplemental figure legends and Table S1. [file mbio.01948-25-s0004.docx]

**Figure Legends**

**Figure S1. The Mtr efflux pump partially mediates the enhancement of *N. mucosa* antibiotic resistance on SCFM2.**

(**A, C, D**) Minimum inhibitory concentrations (MICs) of the indicated antibiotics for the *N. mucosa* wild type (WT) and Mtr efflux mutant (*ΔmtrD*) cultured as a lawn on tryptic soy broth + yeast extract (TSBYE) or synthetic cystic fibrosis medium (SCFM2) agar. Lawns were cultured under oxic conditions since growth was not perceptible under anoxic conditions. Numbers above TSBYE bars: fold change in median comparing WT to *ΔmtrD*. Numbers above SCFM2 bars: fold change in median comparing SCFM2 to TSBYE. Dotted lines indicate the max or minimum MIC value. For azithromycin, plotted MIC values for the *ΔmtrD* mutant are the concentrations at which full (not partial) inhibition occurred.

(**B**) Representative MIC results for azithromycin. Dotted lines indicate the MIC in µg/mL. Left: top dotted line, MIC for WT; bottom dotted line, MIC for *ΔmtrD*. Right: dotted line, MIC for *ΔmtrD*.

(A-D) Data represent ≥2 biological replicates (performed on separate days); gray bars, median.

**Figure S2. Inhibition of *N. mucosa* L-lactate utilization and pyrimidine biosynthesis in synthetic sputum.**

(**A, B**) Growth yields of *N. mucosa* after culture for 24 h under anoxic conditions in (A) tryptic soy broth + yeast extract + 10 mM nitrate (TSBYE) or (B) synthetic cystic fibrosis medium (SCFM2) with increasing amounts of oxalate.

(**C**) Competitive indexes (CI) for the *N. mucosa* wild type (WT) and L-lactate dehydrogenase mutant (*ΔlutC*) after co-culture for 24 h under anoxic conditions in SCFM2 + 10 mM sodium oxalate or an equal volume of H_2_O (-).

(**D, E**) Growth yields of *N. mucosa* after culture for 24 h under anoxic conditions in (D) TSBYE + 10 mM nitrate or (E) SCFM2 + 1 mM PALA (N-phosphonacetyl-L-aspartate) or an equal volume of PBS (-).

(**F**) CI for the *N. mucosa* WT and pyrimidine biosynthesis mutant (*ΔpyrC*) after co-culture for 24 h under anoxic conditions in SCFM2 + 1 mM PALA or an equal volume of PBS (-).

(C, F) CI determined by dividing the output WT:mutant ratio by the input WT:mutant ratio (~1:1).

(A-F) Media were de-oxygenated in (D-F), but not (A-C), prior to inoculation at OD_600_ = 0.001 (~10^5^ total CFU/mL). CFU were necessary to assess growth in SCFM2 due to its turbidity. Data represent ≥2 biological replicates (performed on separate days), each with ≥2 technical replicates (total n = 4-12); gray bars, median; *, P < 0.05; **, P < 0.01; ns, not significant (two-tailed Mann-Whitney test).

**Table S1. Primers used in this study.**

| **Primer target** | **Name** | **Sequence (5’ to 3’)** | **Underlined region** |
| --- | --- | --- | --- |
| EZ-Tn5 transposon | 5Phos-ME-aphA-F | [Phos]CTGTCTCTTATACACATCTTTATACGCTGAATCATCCAATGAC | EZ-Tn5 transposase recognition sequence |
|  | 5Phos-ME-aphA-R | [Phos]CTGTCTCTTATACACATCTGAAGGTGTTGCTGACTCATACC |  |
| Tn-seq library preparation | Nm-Tn-1 | GGTCCACCTACAACAAAGCTCTCATCAACCGTGG |  |
|  | olj376 | GTGACTGGAGTTCAGACGTGTGCTCTTCCGATCTGGGGGGGGGGGGGGGG |  |
|  | Nm-Tn-2 | [Biotin]AATGATACGGCGACCACCGAGATCTACAC[UDI]ACACTCTTTCCCTACACGACGCTCTTCCGATCTCCTGGTATGAGTCAGCAACACC | unique dual index |
|  | BC | CAAGCAGAAGACGGCATACGAGAT[UDI]GTGACTGGAGTTCAGACGTGTG | unique dual index |
| kanamycin resistance gene | kan-f | TTATACGCTGAATCATCCAATGAC |  |
|  | kan-r | GAAGGTGTTGCTGACTCATACC |  |
| *lutC* upstream region | NM96_03080-up-f | ATGCCGTCTGAAGTATTAGGTCTTAGCAGTCATCC | *Neisseria* DNA uptake sequence |
|  | NM96_03080-up-r | GTCATTGGATGATTCAGCGTATAAGCTTTGCCTCCGGTACG | overlaps for DNA assembly |
| *lutC* downstream region | NM96_03080-dn-f | GGTATGAGTCAGCAACACCTTCCTACGCAAACCATCAAGTTCC |  |
|  | NM96_03080-dn-r | ATGCCGTCTGAACCGGACAATGGTTCATACAC | *Neisseria* DNA uptake sequence |
| *ilvB* upstream region | NM96_09260-up-f | ATGCCGTCTGAAGCTGTGTTCGAGTTCGGG | *Neisseria* DNA uptake sequence |
|  | NM96_09260-up-r | GTCATTGGATGATTCAGCGTATAACAAGCCCTCCAAGCAGCG | overlaps for DNA assembly |
| *ilvB* downstream region | NM96_09260-dn-f | GGTATGAGTCAGCAACACCTTCCGACAATGCGACATATCTTATC |  |
|  | NM96_09260-dn-r | ATGCCGTCTGAAGAGGGTAAAGGGTTAAACG | *Neisseria* DNA uptake sequence |
| *pyrC* upstream region | NM96_07040-up-f | ATGCCGTCTGAACCAATGGCAGATTACTCCTG | *Neisseria* DNA uptake sequence |
|  | NM96_07040-up-r | GTCATTGGATGATTCAGCGTATAACGTATGAAAATGCCTGCCCG | overlaps for DNA assembly |
| *pyrC* downstream region | NM96_07040-dn-f | GGTATGAGTCAGCAACACCTTCGTTTCAGACGACCCCGTGATG |  |
|  | NM96_07040-dn-r | ATGCCGTCTGAAGAAGACGGCGTGTTCACGC | *Neisseria* DNA uptake sequence |
| *narG* upstream region | NM96_08885-up-f | ATGCCGTCTGAAACTGTCAAAGCCAAGCCCG | *Neisseria* DNA uptake sequence |
|  | NM96_08885-up-r | GTCATTGGATGATTCAGCGTATAAGGCAGGTTCCTTATGATTGTTATCC | overlaps for DNA assembly |
| *narG* downstream region | NM96_08885-dn-f | GGTATGAGTCAGCAACACCTTCGGCCGTCTGAAAACTTAGTGACG |  |
|  | NM96_08885-dn-r | ATGCCGTCTGAACGGTGCAAATGGCAGTATCG | *Neisseria* DNA uptake sequence |
| *mtrD* upstream region | NM96_08370-up-f | ATGCCGTCTGAACGTCTGAAAACCTGAAACACGG | *Neisseria* DNA uptake sequence |
|  | NM96_08370-up-r | GTCATTGGATGATTCAGCGTATAACCGGTCAGGCTGATGGTG | overlaps for DNA assembly |
| *mtrD* downstream region | NM96_08370-dn-f | GGTATGAGTCAGCAACACCTTCCCAAATCGACAGTTCCAC |  |
|  | NM96_08370-dn-r | ATGCCGTCTGAACACTTCGGAAGCAGCTTTG | *Neisseria* DNA uptake sequence |
